# Small molecule produced by *Photorhabdus* interferes with ubiquinone biosynthesis in Gram-negative bacteria

**DOI:** 10.1128/mbio.01167-24

**Published:** 2024-09-10

**Authors:** Rachel Bargabos, Akira Iinishi, Bryson Hawkins, Thomas Privalsky, Norman Pitt, Sangkeun Son, Rachel Corsetti, Michael F. Gates, Ryan D. Miller, Kim Lewis

**Affiliations:** 1 Antimicrobial Discovery Center, Northeastern University, Boston, Massachusetts, USA; Department of Biochemistry & Biomedical Sciences, McMaster University, Hamilton, Ontario, Canada

**Keywords:** natural antimicrobial products, antibiotic resistance, Gram-negative bacteria

## Abstract

We report the identification of 3,6-dihydroxy-1,2-benzisoxazole (DHB) in a screen of *Photorhabdus* and *Xenorhabdus*, whose symbiotic relationship with eukaryotic nematodes favors secondary metabolites that meet several requirements matching those for clinically useful antibiotics. DHB is produced by *Photorhabdus laumondii* and is selective against the Gram-negative species *Escherichia coli, Enterobacter cloacae, Serratia marcescens, Klebsiella pneumoniae, Proteus mirabilis,* and *Acinetobacter baumannii*. It is inactive against anaerobic gut bacteria and nontoxic to human cells. Mutants resistant to DHB map to the ubiquinone biosynthesis pathway. DHB binds to 4-hydroxybenzoate octaprenyltransferase (UbiA) and prevents the formation of 4-hydroxy-3-octaprenylbenzoate. Remarkably, DHB itself is prenylated, forming an unusable chimeric product that likely contributes to the toxic effect of this antimicrobial. DHB appears to be both a competitive enzyme inhibitor and a prodrug; this dual mode of action is unusual for an antimicrobial compound.

**IMPORTANCE:**

The spread of resistant pathogens has led to the antimicrobial resistance crisis, and the need for new compounds acting against Gram-negative pathogens is especially acute. From a screen of *Photorhabdus* symbionts of nematodes, we identified 3,6-dihydroxy-1,2-benzisoxazole (DHB) that acts against a range of Gram-negative bacteria, including *Escherichia coli*, *Enterobacter cloacae*, *Klebsiella pneumoniae*, and *Acinetobacter baumannii*. DHB had previously been isolated from other bacterial species, but its mechanism of action remained unknown. We show that DHB is unique among antimicrobials, with dual action as an inhibitor of an important enzyme, UbiA, in the biosynthesis pathway of ubiquinone and as a prodrug. DHB is a mimic of the natural substrate, and UbiA modifies it into a toxic product, contributing to the antimicrobial action of this unusual antibiotic. We also uncover the mechanism of DHB selectivity, which depends on a particular fold of the UbiA enzyme.

## INTRODUCTION

The discovery of antimicrobials that target Gram-negative bacteria is an ongoing challenge. A restrictive outer membrane and multidrug resistance (MDR) pumps make up a formidable penetration barrier (1). As a result, the last class of antimicrobials effective against Gram-negative bacteria to reach the clinic were the synthetic fluoroquinolones, developed in the 1960s (2, 3). Gram-negative pathogens such as *Escherichia coli, Klebsiella pneumoniae, Pseudomonas aeruginosa,* and *Acinetobacter baumannii* present particularly challenging cases of multidrug resistance (4). With this in mind, it is important to look beyond traditional antibiotic producers, such as Actinomycetes, which have been extensively mined for the production of antimicrobial natural products (5, 6). For this, we look to underexplored producer organisms such as *Photorhabdus* and *Xenorhabdus*.

*Photorhabdus* and *Xenorhabdus* are unique in their symbiotic relationship with entomopathogenic nematodes. Nematodes feed by invading insect larvae and subsequently releasing gut bacteria into their prey to break down and metabolize the tissue (7, 8). The symbionts then begin to produce antimicrobial compounds to fend off invading bacteria, including those from the nematode gut microbiome, composed primarily of *Burkholderiales (Achromobacter* and *Rheinheimera), Proteobacteria (Pseudomonas), Enterobacteriales (Serratia),* and *Firmicutes (Staphylococcus* and *Bacillus*) (9). *Photorhabdus* and *Xenorhabdus* produce compounds in the presence of their eukaryotic partner that should be non-toxic to their host. Antimicrobials from nematophilic bacteria must be able to disseminate well through insect larvae, suggesting good pharmacokinetics. We recently described several new antibiotics from *Photorhabdus*: the darobactins, a new class of antibiotics that act selectively against Gram-negative bacteria by targeting the BamA chaperone located in the outer membrane (10); dynobactins, which act against the same target (11); and ADG, an inhibitor of transcription (12). Odilorhabdins from *Xenorhabdus* represent a new class of broad-spectrum inhibitors of translation (13).

Differential screening—testing against a target pathogen such as *E. coli* and counter-screening against *Staphylococcus aureus*—allows us to select for antimicrobials of interest and avoid broadly toxic compounds (6). There are distinct advantages to narrow-spectrum antibiotics—they do not disrupt the gut microbiome and do not lead to resistance in off-target bacteria (6, 14, 15). The recent discoveries of darobactin (10), a thanatin derivative (16), lolamicin (17), zosurabalpin (18), and hygromycin A (19) show that narrow-spectrum antibiotics have mechanisms of action that spare the gut microbiome, avoiding secondary infection (20). Herein, we describe the isolation and mode of action of 3,6-dihydroxy-1,2-benzisoxazole (DHB) from *Photorhabdus laumondii* that targets Gram-negative pathogens. The compound was previously reported from extracts of *Chromobacterium* (21) and *Bradyrhizobium denitrificans* (22). It apparently targets quinone synthesis; however, the mechanism of action of this compound had not previously been identified.

**Fig 1:**
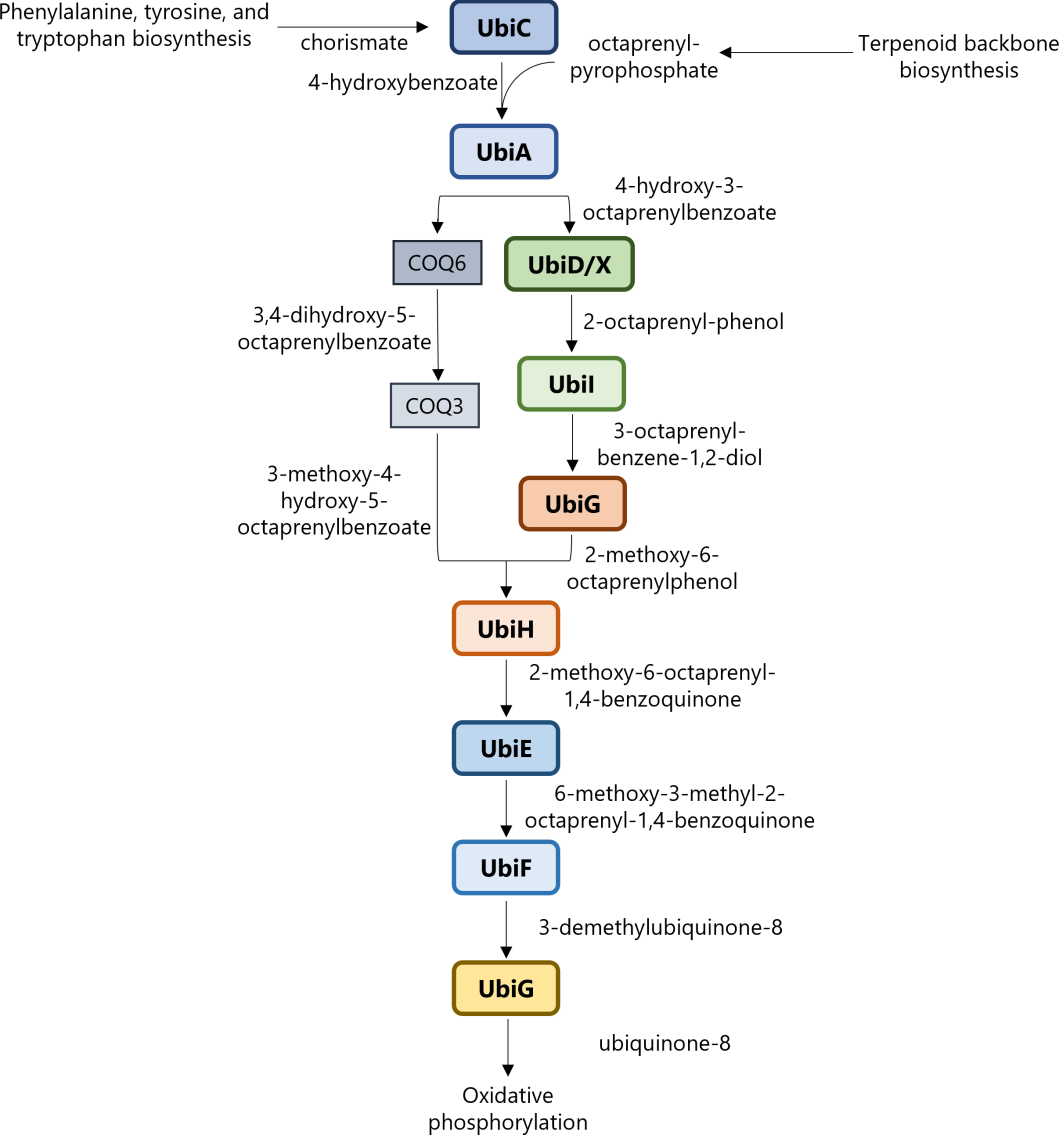
Ubiquinone biosynthesis pathway overview in *E. coli*.

Bacteria use two types of quinones in the respiratory chain, ubiquinone (UQ) and naphthoquinones (menaquinone and demethylmenaquinone). In *E. coli*, UQ is responsible for providing electrons to O_2_ reductases during aerobic respiration, while menaquinone (MK) and demethylmenaquinone (DMK) are primarily used during anaerobic respiration (23, 24). Gram-positive bacteria utilize MK and DMK instead of ubiquinone for aerobic respiration (25, 26). The majority of ubiquinone 8 (UQ_8_) formed aerobically by *E. coli* is made from the precursor 4-hydroxybenzoate (4-HB) (27). Gram-negative bacteria form 4-HB from chorismate, whereas mammalian cells synthesize 4-HB from tyrosine (28, 29). In the early steps of the ubiquinone biosynthesis pathway, chorismate pyruvate-lyase (UbiC) forms 4-HB and pyruvate from chorismate. 4-HB octaprenyltransferase (UbiA) then forms 4-hydroxy-3-octaprenylbenzoate (4-H-3-OPB) from 4-HB and octaprenyl pyrophosphate (Fig. 1). 4-H-3-OPB continues downstream through a series of decarboxylation, hydroxylation, and methylation reactions to become ubiquinone-8 (UQ_8_) (30). In this study, we report that UbiA is the target of DHB and show that this substrate mimic is prenylated by the biosynthetic pathway.

## RESULTS

### Identification of DHB

**Fig 2:**
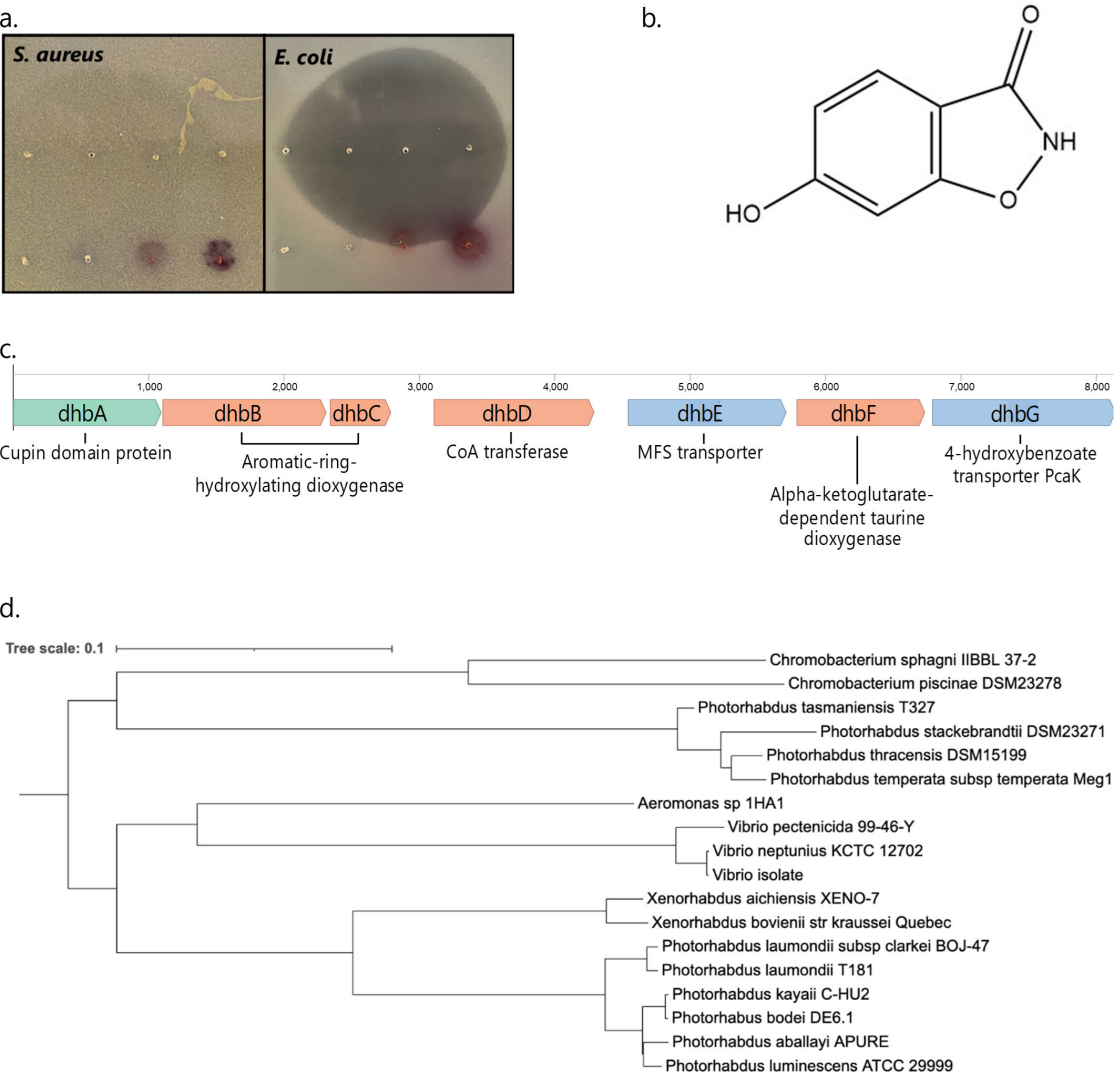
(a) *P. laumondii* grown in liquid culture was tested for growth inhibition of *E. coli* MG1655. Concentrated HPLC fractions produced a zone of inhibition on an *E. coli* but not *S. aureus* lawn. (b) DHB structure. (c) DHB biosynthetic gene cluster with predicted gene function. (d) Phylogeny of the DHB BGC across species. Branch lengths and scale bar represent the average number of nucleotide substitutions per base between sequences.

We screened a library of 183 *Photorhabdus* and *Xenorhabdus* isolates from Thailand for antimicrobial activity. Isolates were tested against several species, including *E. coli, S. aureus, P. aeruginosa*, and *Borreliella burgdorferi*. The supernatant was concentrated at least 20× from the original fermentation to gain access to potential “silent” biosynthetic operons. We focused on an extract from a strain of *P. laumondii,* but initial attempts at isolating an active fraction by organic extraction, resin extraction, or HPLC were not successful. We then turned to anion exchange chromatography. Upon 100× concentration of a fraction eluted at pH 3, we observed a zone of inhibition on a Petri dish overlayed with *Escherichia coli*, but no counterpart zone on plates with *S. aureus* (Fig. 2a). This method of selective screening uses both Gram-negative and Gram-positive pathogens to identify antimicrobials with narrow-spectrum activity and eliminate broadly toxic compounds commonly present in bacterial fermentations. Further bioactivity-guided fractionation against *E. coli* using high-performance liquid chromatography (HPLC) yielded a pure compound with an exact mass of [M + H]^+^ 152.0342 *m/z* and molecular formula C_7_H_6_NO_3_ (calculated [M + H]^+^ 152.0348, Δ3.9 ppm) identified by mass spectrometry (Fig. S1 and S2). The chemical structure was determined with the use of a ^1^H NMR spectrum showing the presence of three aromatic protons (Fig. S3). The antimicrobial was determined to be 3,6-dihydroxy-1,2-benzisoxazole, a compound originally isolated in 1983 from *Chromobacterium* and again in 2021 from *Vibrio* (previously annotated as *Bradyrhizobium*) (21, 22) (Fig. 2b).

### Identification of the biosynthetic gene cluster

In order to determine the biosynthetic gene cluster (BGC) for DHB, we took advantage of several different producers, looking for a shared BGC. Targeted LC-MS detected DHB production from three strains of *Photorhabdus laumondii* in addition to a *Vibrio* isolate (22): *P. laumondii* T181, *P. laumondii* TT01, and an environmental isolate *P. laumondii* S56. No detectable production was observed from closely related species *P. laumondii* subsp*. clarkei* DSM 105531, *Photorhabdus kayaii* DSM 15194, *Photorhabdus kleinii* DSM 23513, or *Photorhabdus noenieputensis* DSM 25462. Candidate genes that were present in DHB producers and absent in non-producer strains were identified using UniProtKB BLAST annotations and top hits. Considering that DHB production from “non-producer” strains might be below the limit of LC-MS detection, a second genomic review was conducted, expanding the search to cases where at least two “rare” genes, present in the three producer strains and at most one non-producer strain, were co-clustered. This analysis yielded one candidate BGC for the biosynthesis of DHB (Fig. 2c).

The composition of the BGC is consistent with DHB biosynthesis. Two synthetic routes are possible: 4-HB or salicylic acid could be oxygenated by the DhbBC complex to produce 2,4-dihydroxybenzoic acid, followed by ring closure and N-O bond formation by DhbD and DhbF to form DHB (Fig. 2c; Fig. S4). Alternatively, based on sequence homology, DhbBC may be an aromatic-ring-hydroxylating dioxygenase, in which case it would instead act on 4-HB to produce 4,5,6-trihydroxycyclohexa-1,3-diene-1-carboxylic acid, which could undergo the same ring closure and N-O bond formation by DhbD and DhbF. This pathway would then require the elimination of the 3-hydroxy to form DHB, which might be catalyzed by an unknown enzyme or occur spontaneously. Notably, this BGC has a GC content of 35.6%, in comparison to the *Photorhabdus* genome content of 42.5%–42.7% (31), suggesting acquisition by horizontal gene transfer. This is not uncommonly seen in *Photorhabdus*; the antibiotic darobactin A is produced by *Photorhabdus khanii* and has a BGC with a GC content of 32% (10), and the *Photorhabdus australis-*produced dynobactin BGC sits at 34% (11). The phylogenetic relatedness of this BGC is shown (Fig. 2d). Interestingly, for most genes in the BGC, the percent identity to *P. laumondii* T181 is the same or higher for *Vibrio* than for *Photorhabdus stackebrandtii* (Table S1). Despite *Chromobacterium* being the most distantly related genus, its BGC is the closest relative to a group of *Photorhabdus*. This is evidence against vertical gene descension, which would show percent identity tracked with species relatedness, and suggests there may be two distinct points where the cluster was acquired by *Photorhabdus*.

### Potency and spectrum of action

DHB is active against a range of Gram-negative pathogens and inactive against Gram-positive pathogens and relevant gut bacteria (Table 1; Fig. S5). Based on this, DHB shows activity under aerobic conditions and a lack of off-target activity. Notably, DHB is inactive against *Bacteroides*, the main group of Gram-negative gut symbionts. DHB is active against enteric bacteria, such as *E. coli, Enterobacter cloacae,* and *K. pneumoniae* only under aerobic conditions, where they can cause clinically relevant systemic infections. DHB is also non-toxic to the three different human cell lines that were tested. Additionally, identical MICs to wild-type *E. coli* MG1655 and *E. coli* WO153, a leaky outer membrane strain with a mutant *asmB1* allele and *ΔtolC* efflux pump, indicate that DHB potency is not affected by changes in the permeability of the outer membrane, signifying it can penetrate the outer membrane.

**TABLE 1:** Spectrum of activity^*a*^

Organism	DHB MIC (μg mL^−1^)
Pathogenic bacteria	
*Escherichia coli* MG1655	1
*Escherichia coli* ATCC 25922	1–2
*Escherichia coli* WO153 *asmB1*, *ΔtolC*	1
*Enterobacter cloacae* ATCC 13047	0.5
*Serratia marcescens* ATCC 13880	1–2
*Klebsiella pneumoniae* BAA-2146	2
*Klebsiella pneumoniae* BAA-43816	4
*Proteus mirabilis* HI4320	2–8
*Acinetobacter baumannii* ATCC 19606	16
*Pseudomonas aeruginosa* PAO1	>128
*Staphylococcus aureus* HG003	>128
Gut anaerobes	
*Bacteroides uniformis* KLE 1601	>128
*Eggerthella lenta* KLE 2234	>128
*Bifidobacterium bifidum* KLE 2535	>128
*Veillonella ratti* KLE 2366	64
*Ruminococcus gnavus* ATCC 29149	>128
*Clostridium hathewayi* KLE 1709	>128
*Enterococcus faecalis* KLE 2341	>128
Human cell lines	
HepG2	>128
FaDu	>128
HEK293	>128

^*a*^ Bacteria were cultured in Mueller-Hinton II broth, and minimum inhibitory concentration (MIC) was determined in duplicate by broth microdilution in microtiter plates. For anaerobes, bacteria were cultured in duplicate in Gifu Anaerobic Broth in an anaerobic chamber. For cytotoxicity, cells were grown in triplicate in Eagle’s minimum essential media, and viability was determined by resazurin cell viability assay.

**Fig 3:**
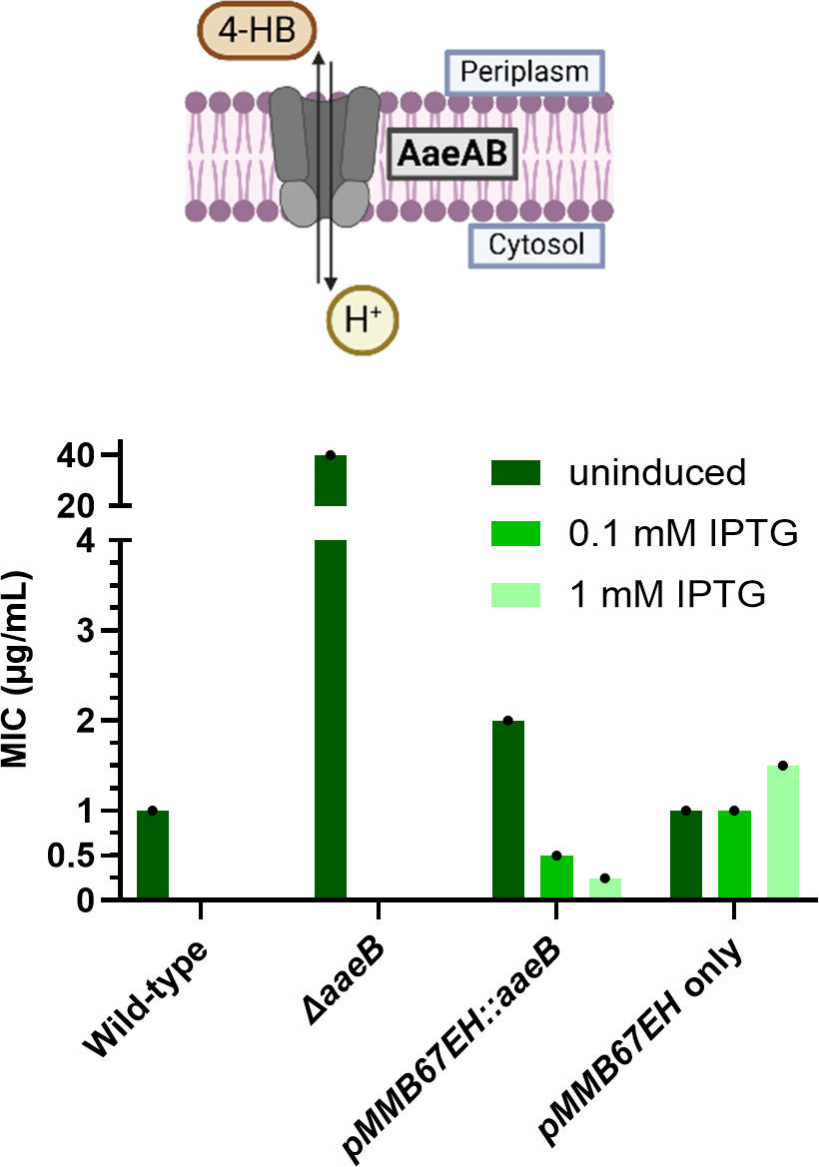
Competition between DHB and 4-HB. 4-HB is pumped out of the cell by aromatic carboxylic acid efflux pump AaeAB to prevent toxicity from buildup of the intermediate. 4-HB is antagonistic to DHB activity. Increased inhibition of DHB was observed when 4-HB levels were low due to pump overexpression. MIC is measured in duplicate, and SD is shown.

The precursor 4-HB was reported to be antagonistic to DHB (22). In order to determine this antagonism quantitatively, we performed a checkerboard assay. The antagonism was obvious, with an FIC value of 8 (Fig. S6). We reasoned that DHB potency could be affected by the efflux of 4-HB by the AaeAB (YhcQP) aromatic carboxylic acid efflux pump. Addition of 4-HB to *E. coli* leads to the upregulation of AaeAB, responsible for controlling intracellular levels of the toxic intermediate (32–34) (Fig. 3). We reasoned that overexpression or knockout of the pump will decrease or increase the amount of 4-HB in the cell, respectively, resulting in changes in *E. coli* sensitivity to DHB. *AaeB* was cloned into an isopropyl β-D-1-thiogalactopyranoside (IPTG)-inducible plasmid *pmmB67EH* for overexpression, and *ΔaaeB* and *ΔtolC* strains were made using lambda red recombination in *E. coli* MG1655. Induction of the *aaeB* overexpression strain with increasing amounts of IPTG caused a drop in the MIC from 2 to 0.25 µg/mL as 4-HB was pumped out of the cell. Conversely, *ΔaaeB* required four- to sixfold more DHB (16–64 μg/mL) for inhibition (Fig. 3; Table S2). This suggests competition between 4-HB and DHB for the UbiA catalytic sites since increased amounts of 4-HB trapped in *ΔaaeB* cells appeared to outcompete DHB and permitted cell growth. DHB was previously found to have greater activity in minimal media, with *E. coli* MICs between 0.25 and 0.5 µg/mL, likely because 4-HB may not always be in excess in nutrient-poor conditions (22). Reported DHB potencies in the previous literature range from <1 µg/mL (21) to >500 µg/mL (22) in Mueller-Hinton broth against strains of *E. coli*. We observed a loss of DHB potency upon the addition of glucose, a known repressor of *ubiA* transcription (35, 36), to the growth media (Table S3). Differing amounts of 4-HB and glucose in the growth media and varying methods of MIC testing could explain these discrepancies.

### Mechanism of action

4-hydroxybenzoate is a precursor of ubiquinone biosynthesis and resembles DHB. 4-HB was shown to antagonize DHB, pointing toward a target in the ubiquinone biosynthesis pathway (22) (Fig. 1). Molecular modeling suggested binding to chorismate pyruvate lyase (UbiC) (22); however, the target has not been experimentally determined.

### Selection for resistant mutants

**Fig 4:**
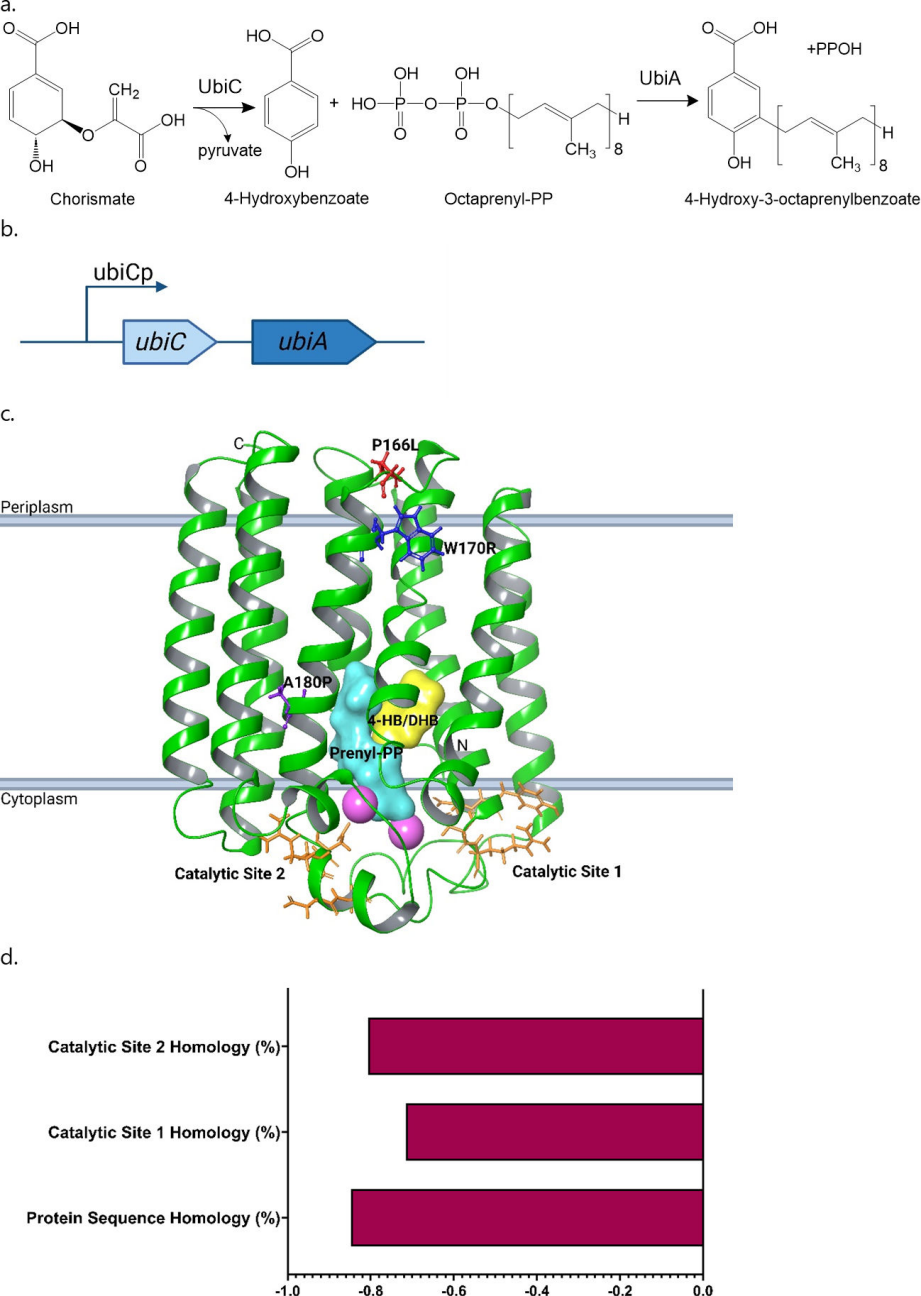
(a) Ubiquinone biosynthesis pathway in *Escherichia coli*. Chorismate is converted to 4-hydroxybenzoate by chorismate pyruvate-lyase (UbiC), followed by the formation of 4-H-3-OPB catalyzed by 4-HB octaprenyltransferase (UbiA). (b) Oriented side view of UbiA for *E. coli* generated via RosettaCM, PDB ID:4OD5, single-point resistant mutations mapped at amino acid positions 166 (red), 170 (blue), and 180 (purple) onto substrate bound UbiA, 4-HB (yellow), and prenyl-pyrophosphate (cyan), Mg^2+^ co-factors and aspartic acid-rich catalytic regions shown in pink and orange, respectively. (c) UbiAC operon. (d) Pearson correlations of MIC with full protein sequences of UbiA and its catalytic sites of species susceptible to DHB. All values are <−0.5, indicating a strong negative correlation.

In order to identify the target of DHB, we selected for resistant mutants. *E. coli* MG1655 at increasing cell densities was plated on DHB. Slow-growing colonies of *E. coli* began to appear on plates containing DHB at 4× MIC after 3–5 days. MICs of the mutants were three- to fourfold greater than wild-type *E. coli* (Table 2). Full genome sequencing and variant calling performed using breseq revealed mutations within the ubiquinone biosynthesis pathway, specifically, *ubiC*, *ubiA*, and the intergenic region between these genes (Table 2) (Fig. 4a and b). Mutant 1 had delayed growth, and mutant 4 plateaued in mid-exponential phase growth in comparison to wild-type *E. coli*. The mutation frequency was 1.8 × 10^−9^ 3 days after antibiotic selection. DHB was previously seen to be inactive against *E. coli* grown anaerobically with and without glucose, as well as respiratory-deficient strain *ΔhemB* (37), and we confirmed this observation (Table S3). Mutations within a pathway essential for aerobic growth were consistent with these observations. Point mutations in UbiA in three mutants caused structurally significant amino acid changes (A180P, W170R, and P166L) that likely affected the conformation of the alpha helices (Fig. 4c). Tryptophan and proline amino acid changes especially affect alpha helices, as they favor different helical conformations (38). Single nucleotide polymorphisms in intergenic regions have been shown to influence the expression of nearby genes (39, 40). In another mutant, insertion of the 1,338-bp IS186 mobile element with deletions and duplications on both the left and right margins disrupts UbiC, a much smaller (498 bp) protein. This disruption likely results in decreased expression of *ubiA* and reduced DHB potency. Complete gene deletions of both potential targets were then created. The MIC of DHB in *ΔubiC* was similar to the parental strain, while no inhibition of *ΔubiA* by DHB was observed. Additionally, overexpression of *ubiA* results in a small decrease in DHB MIC (Fig. S7). From this, we determined that *ubiA* is the sole target of DHB (Table 3). The increase in susceptibility to an antibiotic upon target overexpression is the opposite of what is usually expected and indicates the possible formation of a toxic product.

**TABLE 2:** Mutants with decreased susceptibility to DHB

*Escherichia coli* MG1655 mutants		DHB MIC (μg mL^−1^)
Location	Mutation	
*Escherichia coli* MG1655 (parental)	N/A	1
*ubiA*180	Non-synonymous substitution Alanine → Proline	16
Intergenic (+3/–10)	Substitution G → A	8
*ubiA*170	Non-synonymous substitution Tryptophan → Arginine	16
*ubiA*166	Non-synonymous substitution Proline → Leucine	8
*ubiC* (140–142/165)	Insertion of mobile element Δ2bp :: IS186 + 8 bp :: Δ1bp	8–16

**TABLE 3:** MICs of DHB against *E. coli* lacking *ubiA* and *ubiC*

*Escherichia coli* MG1655 strain	DHB MIC (μg mL^−1^)
*Escherichia coli* MG1655	1
*ΔubiC*	2
*ΔubiA*	>128

Deletion of *ubiA* eliminates UQ_8_ biosynthesis (23). Ubiquinone is responsible for the downstream oxidation of NADH, which results in products NAD^+^ and ubiquinol (QH_2_). The two-electron transfer to ubiquinone results in the donation of two local protons, forming QH_2_ and triggering proton pumping (41, 42). Treatment with DHB resulted in a decrease in the membrane potential measured by DiOC_2_(3) (Fig. S8).

UbiA contains two catalytic pockets with active site residues (Asp191 and Arg72) that are necessary for binding 4-HB (43). *Escherichia coli, Enterobacter cloacae, Klebsiella pneumoniae, Serratia marcescens, Proteus mirabilis,* and *Acinetobacter baumannii* have highly conserved catalytic sites (Table S4) that are correlated with DHB potency (Fig. 4d).

### *ubiA* swap

The ubiquinone biosynthesis pathway in Gram-negative bacteria begins with the formation of 4-HB and pyruvate from chorismate by UbiC (Fig. 4a). UbiA catalyzes the addition of an octa-prenyl tail to form 4-H-3-OPB from 4-HB and octaprenyl-pyrophosphate, which continues downstream to become ubiquinone-8 (UQ_8_) (Fig. 1) (30). Deletion of *ubiA* stops the production of UQ_8_ and disrupts the downstream electron transport. UbiA active site residues (Asp191 and Arg72) are essential for 4-HB binding (43) and highly conserved among *Escherichia coli, Enterobacter cloacae, Serratia marcescens, Proteus mirabilis, Klebsiella pneumoniae,* and *Acinetobacter baumannii*. UbiA structure and active site homology between these strains correlate with DHB potency (Fig. 4d; Table S4). *Pseudomonas aeruginosa* also has significant homology with *E. coli* UbiA and its catalytic residues but displays no susceptibility to DHB. Based on these correlations, we reasoned that *P. aeruginosa* may possess a UbiA that is susceptible to DHB when placed in a different background strain.

**Fig 5:**
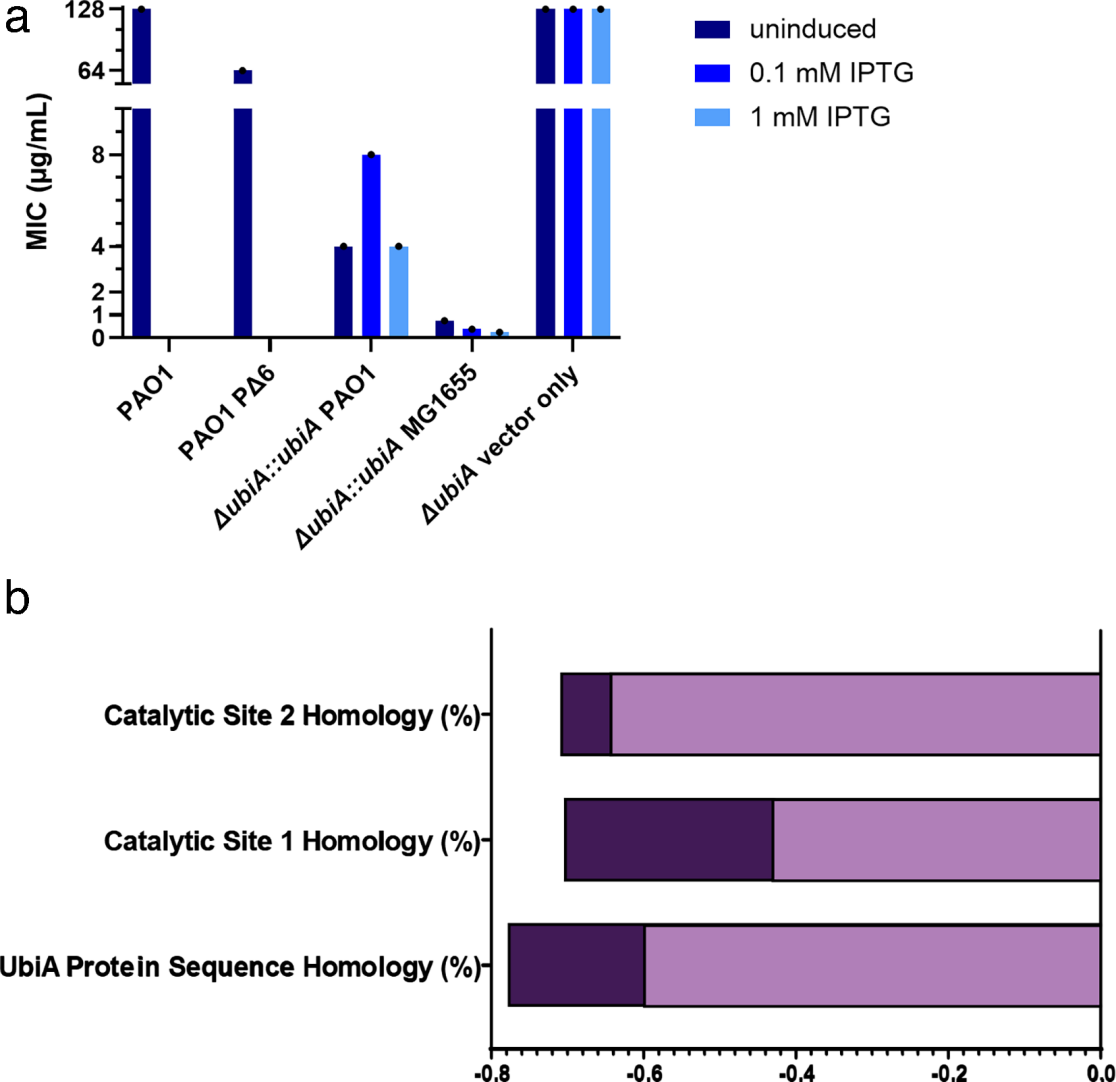
(a) *ubiA* from *E. coli* MG1655 and *P. aeruginosa* PAO1 were complemented into *E. coli* MG1655 *ΔubiA*. IPTG was used for the induction of plasmid pMMB67EH. *ubiA* from MG1655 and PAO1 have 53.45% sequence identity. MIC is measured in duplicate, and SD is shown. (b) Pearson correlations of UbiA and catalytic site homologies to MIC, including *P. aeruginosa* PAO1 MIC of >128 µg/mL (light purple). Updated correlations substituting the MIC of *ΔubiA::ubiA* PAO1 for *P. aeruginosa* PAO1 (dark purple). All correlations are now <−0.5, indicating negative linear correlation.

In order to determine whether differences in the structure of UbiA were directly responsible for the differences in DHB potency among Gram-negative pathogens, we swapped homologous *ubiA* genes between sensitive and resistant species. *E. coli* MG1655 (MIC, 1 µg/mL) and *P. aeruginosa* PAO1 (MIC, 128 µg/mL) were selected for the swap. MIC testing of *Pseudomonas* PΔ6 (MIC, 64 µg/mL), an MDR pump knockout (44), showed a negligible shift in MIC from the wild-type strain (128 µg/mL), suggesting that efflux was not responsible for the low potency of DHB. Additionally, MIC testing of *P. aeruginosa* PAO1-Pore (MIC, 128 µg/mL), with IPTG-inducible hyperporination of the outer membrane (44), indicates that outer membrane integrity has little effect on DHB potency (Fig. S5). A codon-optimized *P. aeruginosa* PAO1 *ubiA* (*ubiA*-PAO1) was inserted into plasmid *pmmb67EH* under an IPTG-inducible promoter and transformed into *E. coli* MG1655 *ΔubiA* cells. As a control, *E. coli* MG1655 *ubiA* (*ubiA*-MG1655) was inserted on the *pmmB67EH* plasmid and complemented into *E. coli ΔubiA* cells. The MIC of the *E. coli ΔubiA::ubiA-*MG1655 strain gradually decreased from 1 to 0.25 µg/mL as increasing amounts of IPTG were added to induce the expression of UbiA (Fig. 5a; Table S5). Complementation with *E. coli ubiA* restored DHB susceptibility of DHB-resistant *ΔubiA*. When the same was done with *ubiA*-PAO1 in *E. coli* MG1655 *ΔubiA*, the MIC remained at 4–8 μg/mL as IPTG was added. The *ΔubiA::ubiA-*PAO1 MIC of 4–8 μg/mL falls between *K. pneumoniae* and *A. baumannii* in both MIC and UbiA protein sequence homology (Table S4). With all strains now displaying consistent correlations, we can conclude that DHB potency is correlated with UbiA sequence identity and catalytic site homology (Fig. 5b; Fig. S9). Given that no change in MIC was observed in *P. aeruginosa* strains with compromised outer membrane, the inactivity of DHB against wild-type *P. aeruginosa* can likely be explained by the flexible metabolism and highly branched respiratory chain of PAO1, which uses a variety of electron donors and acceptors during respiration (45).

Following target identification, we hypothesized that DHB may function as a competitive inhibitor of 4-HB. Computational modeling of the docking was performed using Maestro (version 13.7.125) by Schrödinger to determine the binding energetics of DHB and 4-HB in *E. coli*. A docking grid was generated from an enriched *ab initio* protein model based on the *Aeropyrum pernix* K1 crystal structure (PBD: 4OD5, respectively). Prior to the creation of the grid, inactive geranyl-S-thiolodiphosphate, used in the crystallization process to stop the reaction from proceeding, was converted to reactive geranyl-S-pyrophosphate (GPP). Redocking 4-HB (XP, Glide) showed remarkable accuracy to the original crystallized pose, with an RMSD of 0.15 Å^3^ validating the docking grid. Following this, DHB was docked, showing modestly better binding than 4-HB (−7.1 and −6.3 kcal/mol, respectively). The energetic difference was confirmed using molecular dynamics (see Materials and Methods). Equilibrium frames (200–2,000) were used for molecular mechanics with generalized Born and surface area solvation (mm/gbsa) calculations of the ligand in the active site. Once again, it was evident that DHB has improved binding in the UbiA catalytic region [−30.5 (2) vs −24.1 (5) kcal/mol] compared to 4-HB. The rate-limiting step in the prenylation reaction was the formation of a carbocation created by pyrophosphate cleavage on GPP, forming an unstable intermediate (46). These results followed previous observations that more aromatic ligands have increased susceptibility to attack by the carbocation and formation of the prenylated product. Throughout the simulation, we observed that DHB was closer to the catalytic domain and C1 of GPP, indicating a preference for a pre-reaction state defined by Yang et al. (46). Based on the preferred energetic state of the UbiA catalytic domain, it is predicted that DHB outcompetes 4-HB in both binding and reaction kinetics.

**Fig 6:**
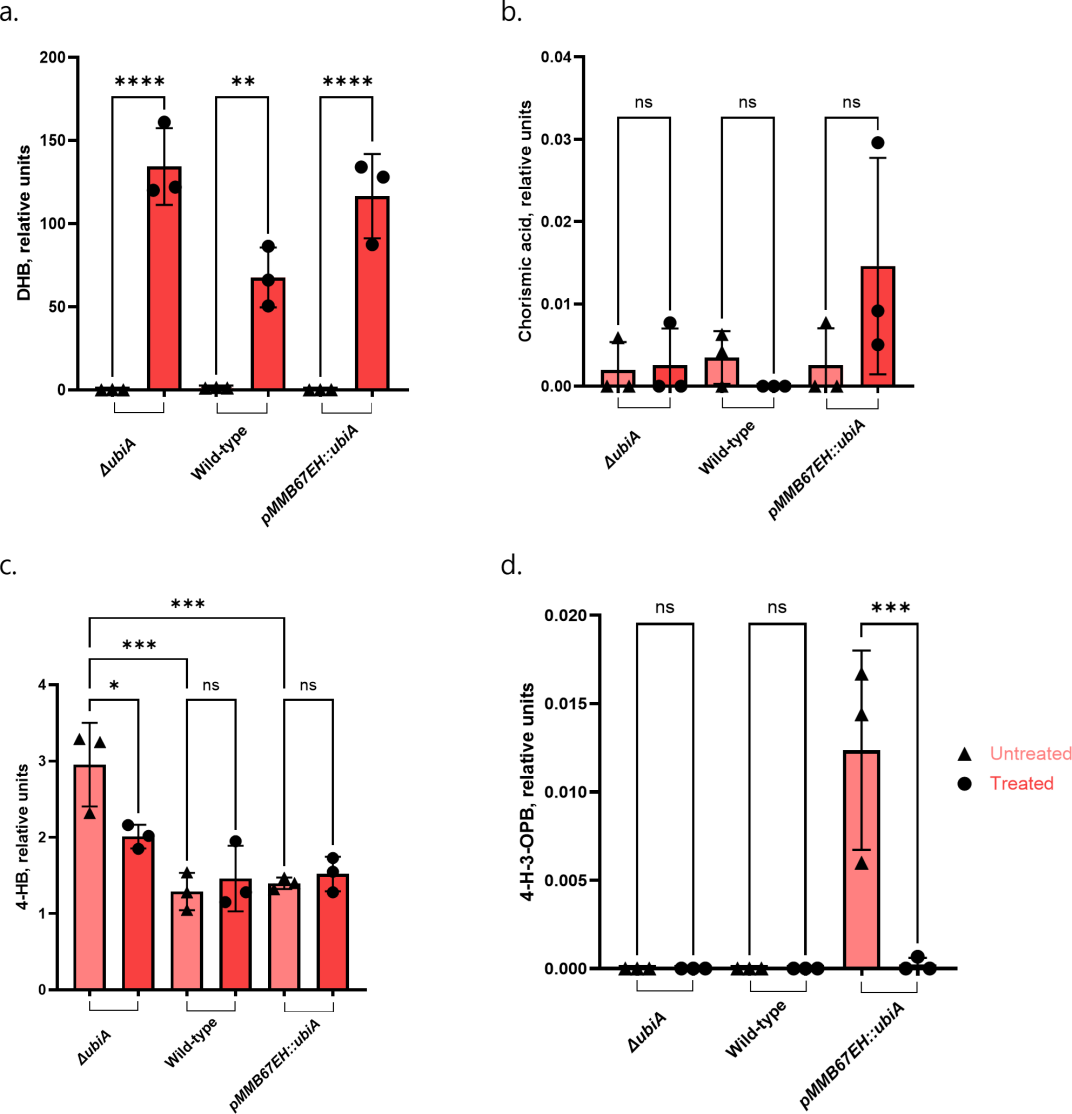
Metabolome profiles of wild-type *E. coli* MG1655, *ubiA* overexpression construct (*ubiA::pmmB67EH*), and *ubiA* knockout (*ΔubiA*) untreated and treated with DHB at 10× MIC. Relative units on the *y*-axis are area normalized by the internal standard, ^13^C_6_ 4-OH-benzaldehyde. Mean amounts and SD of (a) DHB, (b) chorismic acid, (c) 4-hydroxybenzoate, and (d) 4-hydroxy-3-octaprenylbenzoic acid for biological replicates of all three strains shown. Adjusted *P*-values were calculated using the ordinary one-way ANOVA method followed by a Tukey’s *post hoc* test.

The binding of DHB to the UbiA catalytic domain would result in a lack of 4-H-3-OPB, the product of 4-HB and octaprenyl pyrophosphate (Fig. 1 and 4a). Metabolomic profiles of *ΔubiA*, wild-type *E. coli* MG1655, and *pmmB67EH::ubiA* were analyzed for compounds of interest. Metabolome extracts were collected before and after 1 hour of treatment with DHB and analyzed by LC-MS at the Harvard Center of Mass Spectrometry (47) for masses of relevant products and precursors. DHB as a positive control was only detected in treated cells (Fig. 6a), and no statistically significant difference was found in 4-HB precursor chorismic acid in any strains (Fig. 6b), suggesting that UbiC continues to function normally. 4-HB was present in higher amounts in *ΔubiA* than either the overexpression or wild-type strains, indicating it could not be processed by UbiA in the next step of quinone biosynthesis (Fig. 6c). UbiA product 4-H-3-OPB was only seen in the untreated *ubiA* overexpression strain and disappeared when cells were treated with DHB (Fig. 6d). These data show that DHB blocks the formation of the natural product by UbiA.

**Fig 7:**
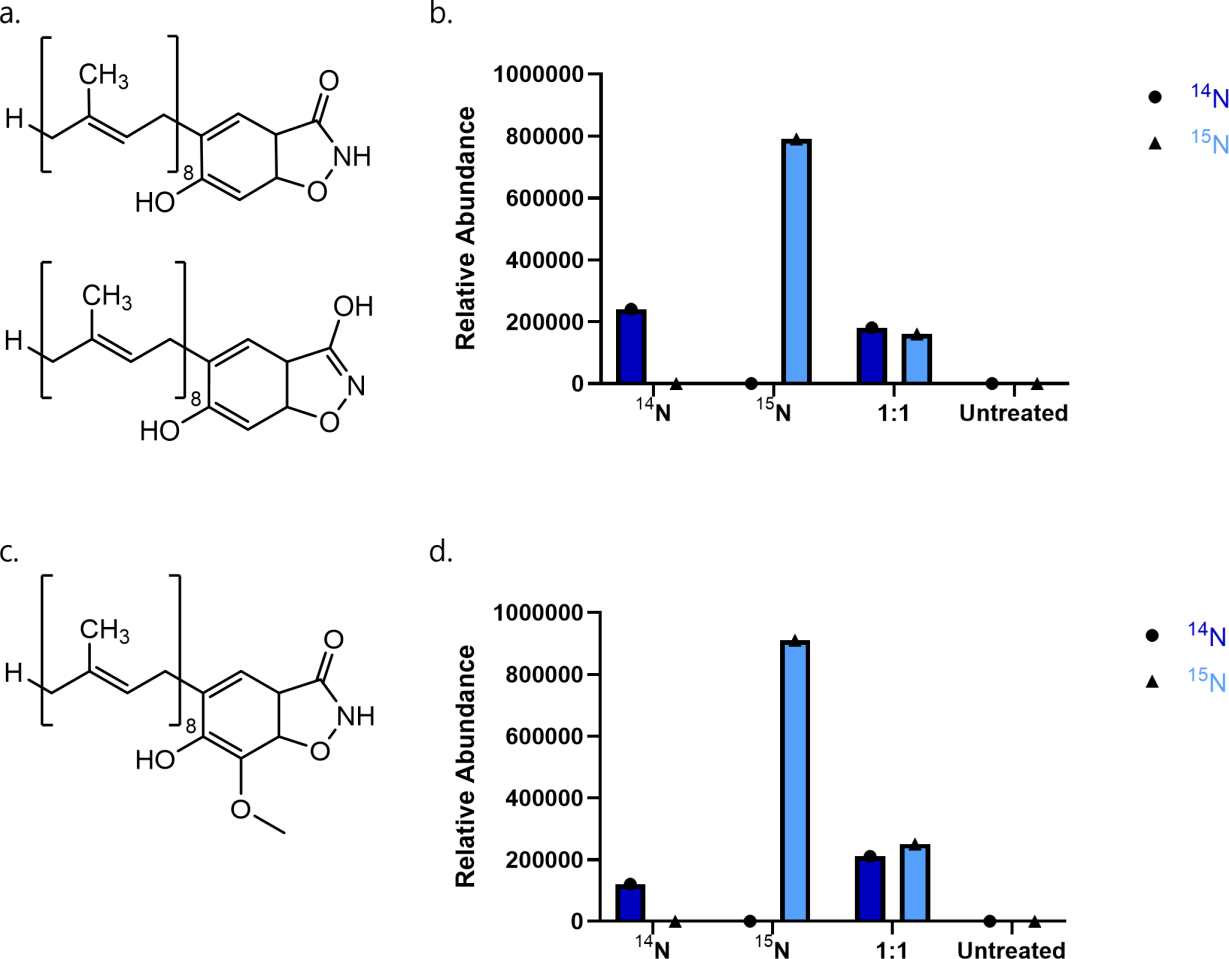
(a) Predicted structure of DHB with the addition of an 8-unit prenyl tail (DHB_8_). (b) Relative abundances of ^14^N and ^15^N DHB_8_ in ^14^N, ^15^N, 1:1 ^14^N:^15^N DHB treated, and untreated *E. coli ubiA* overexpression cells. (c) Predicted structure of 4-octaprenyl-7-methoxy-DHB (O-methylated DHB). (d) Relative abundances of labeled and unlabeled 4-octaprenyl-7-methoxy-DHB in treated and untreated samples.

**Fig 8:**
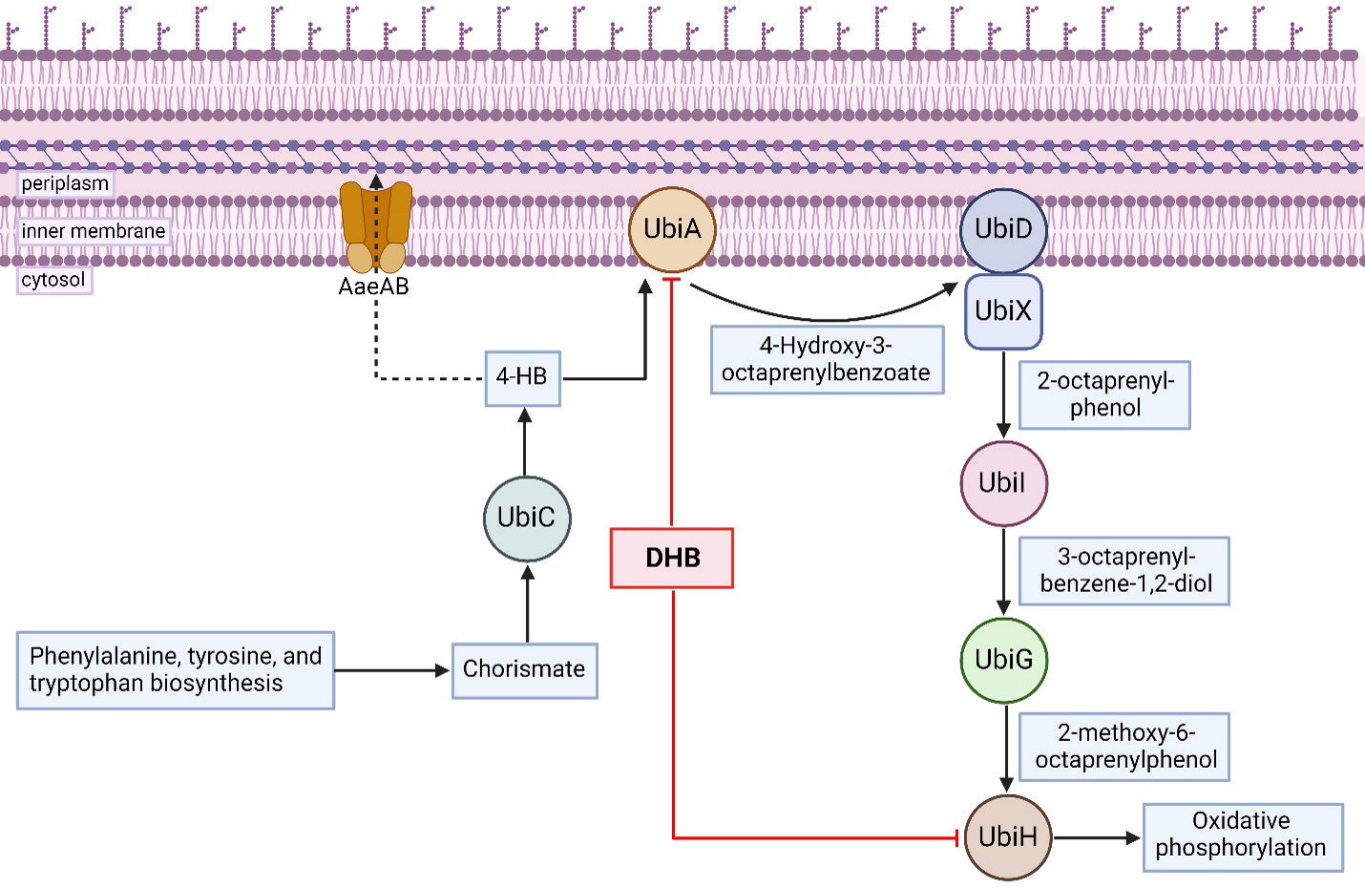
Pathway overview. Prenylation of 4-HB in *E. coli* is stopped when DHB inhibits UbiA. Modified DHB continues downstream until it becomes unusable. Excess 4-HB is pumped out of the cell via aromatic carboxylic acid efflux pump AaeAB.

Molecular modeling suggests that DHB is likely to interact with GPP in the UbiA catalytic pocket. Given that DHB is a mimic of the natural substrate, we considered an intriguing possibility that this antimicrobial compound is modified in the same way as 4-HB and continues downstream in the ubiquinone biosynthesis pathway. We first examined an untargeted metabolome profile for the presence of the predicted structure of prenylated DHB (DHB_8_) (Fig. 7a). Since a reference standard was not available, however, we could not be sure that the mass seen in the untargeted analysis was truly a DHB-based product. To address this, we used a ^15^N-labeled version of DHB in addition to the normal treatment with unlabeled (^14^N dominant) DHB. The mass shift from ^15^N to ^14^N is visible in the mass spectra and would indisputably indicate the presence of DHB in the observed product. The *ubiA* overexpression strain was treated with ^14^N DHB, ^15^N DHB, and a 1:1 ratio of ^14^N:^15^N DHB for 1 hour, then lysed and analyzed by mass spectrometry. Treatment with the isotopic mix creates a visible shift of one atomic mass unit for direct comparison within a single sample. Samples were run on a C18 column with a selected ion monitoring (SIM) method that focused on gathering data at predicted masses of interest. This allowed for increased sensitivity in detecting the potentially low signal of DHB_8_ candidates. Using this method, we observed the predicted mass of the ^14^N prenylated product (698.55 *m/z*) in the ^14^N treated sample, the ^15^N labeled prenylated drug at 699.55 *m/z* in the ^15^N treated sample, and both the labeled and unlabeled compound masses in a 1:1 ratio in the mixed sample (Fig. 7b). The detection of all predicted masses matched the expected pattern, confirming the presence of DHB_8_. We hypothesized that this product could continue in the ubiquinone biosynthesis pathway until the structural differences between DHB and 4-HB make further modifications impossible. In typical *E. coli* ubiquinone biosynthesis, 3-octaprenyl-4-hydroxybenzoate decarboxylase (UbiD) and flavin prenyltransferase (UbiX) interact to catalyze the decarboxylation of 4-hydroxy-3-octaprenylbenzoate. Newly formed 2-octaprenyl-phenol is then hydroxylated by 2-octaprenylphenol 6-hydroxylase (UbiI) to form 3-octaprenyl-benzene-1,2-diol. S-adenosyl-L-methionine-dependent O-methyltransferase (UbiG) then catalyzes its first O-methylation reaction, resulting in 2-methoxy-6-octaprenylphenol (Fig. 1). Metabolomes of drug-treated cells were searched for hydroxylated and O-methylated DHB using the same ^14^N and ^15^N mass differential to confirm the presence of the drug in any downstream products. O-methylated DHB_8_ (alternatively, 4-octaprenyl-7-methoxy-DHB) (Fig. 7c) was detected with the correct pattern of ^14^N and ^15^N, providing strong evidence that the downstream product is present and contains DHB (Fig. 7d). Hydroxylated DHB_8_ was detected in the 1:1 treated sample, but not in individually labeled and unlabeled treated samples. After the O-methylation step, further modification of the drug becomes impossible, as 2-octaprenyl-6-methoxyphenol 4-hydroxylase (UbiH) has no free carbon for a hydroxylation reaction, and no further products containing DHB were detected. The ubiquinone biosynthesis pathway modifying DHB, therefore, must be arrested at this point (Fig. 8). The modified DHB products are likely toxic, contributing to growth inhibition. This would explain why overexpression of *ubiA* results in increased susceptibility to DHB.

## DISCUSSION

DHB was originally isolated from *Chromobacterium violaceum* and discovered to have activity against select Gram-negative pathogens in 1983 (21). Rediscovery in 2021 from a *Vibrio* isolate (22) prompted further exploration. Authors hypothesized that DHB either targeted chorismate pyruvate lyase (UbiC) and shut down the production of 4-HB or competitively bound 4-HB octaprenyltransferase (UbiA), arresting the ubiquinone biosynthesis pathway. The former was evidenced by molecular docking of DHB to UbiC, and the latter by 4-HB antagonism to DHB. We observed similar antagonism between 4-HB and DHB and, in addition, showed that expression of the 4-HB efflux transporter AaeAB increases the potency of DHB. Mutations resistant to DHB mapped to UbiA. Our docking analysis shows that DHB binds well to UbiA, and the resistant mutations diminish the interaction of the antibiotic with the target. Finally, deletion of UbiC had no effect on the potency of DHB. Metabolomic analysis showed the loss of UbiA product 4-hydroxy-3-octaprenylbenzoic acid (4-H-3-OPB) when cells overexpressing *ubiA* were treated with DHB. Together, these data show that DHB is a competitive inhibitor of UbiA. Remarkably, we find that DHB is a mimic of 4-HB, and apart from inhibiting the enzyme, it also serves as a substrate. The *E. coli* metabolome revealed that UbiA catalyzes the addition of an octaprenyl tail to DHB (DHB_8_). Modification of DHB by UbiA is consistent with the known promiscuity of the enzyme. UbiA is known to accept a variety of 4-HB derivatives and related compounds with few requirements, including an electron donor and hydrogen bond donor in *para* position (48, 49).

In the same metabolomic analysis, the O-methylated product of UbiG was found to contain DHB, suggesting the drug can be modified by at least two more enzymes in the pathway. It is possible that the prenylated forms of DHB contribute to the toxic action of the compound against bacteria. Indeed, overexpression of *ubiA* increases the potency of DHB, consistent with the formation of a toxic product. DHB appears to be both an enzyme inhibitor and a prodrug; this dual mode of action is unlike other prodrugs, such as isoniazid, which can function as inhibitors only after modification. Further research will be required to evaluate the potential toxicity of DHB_8_.

DHB is of particular interest due to its high level of selectivity. *Photorhabdus* producers are responsible for several interesting narrow-spectrum antibiotics discovered in recent years (10–12, 50). Much of the selectivity of DHB is owed to the structural features of UbiA and its catalytic sites. In closely related species of Gram-negative Gammaproteobacteria (*E. coli, E. cloacae, K. pneumoniae, S. marcescens, P. mirabilis,* and *A. baumannii*), potency of DHB correlates with the homology of UbiA to the *E. coli* enzyme. The human homolog COQ2 has 32.76% sequence identity with *E. coli* UbiA and is not susceptible to DHB (Fig. S7). Gram-positive bacteria utilize menaquinone and demethylmenaquinone instead of ubiquinone for aerobic respiration (25, 26), do not have UbiA, and, therefore, are not susceptible.

The Gram-negative specificity and the formation of DHB chimeric products are intriguing subjects to explore in future studies. Based on the horizontally acquired nature of DHB and many other antimicrobials produced, *Photorhabdus* and *Xenorhabdus* almost certainly contain other interesting and selective compounds that are waiting to be discovered.

## MATERIALS AND METHODS

### Initial screening

A selection of isolates that had shown promising activity in the past were chosen from a larger library of *Photorhabdus* and *Xenorhabdus* provided by Dr. Aunchalee Thanwisai from Thailand. Strains were struck from cryostocks onto tryptic soy agar (TSA) plates and allowed to grow static at 28°C for 2 days. Bacteria were inoculated into 5 mL overnight cultures of tryptic soy broth (TSB) and grown by shaking at 28°C and 220 rpm. A panel of media consisting of TSB (Sigma-Aldrich), LB (Sigma-Aldrich), nutrient broth (Sigma-Aldrich), TNM-FH insect medium (Sigma-Aldrich), R4, starch, yeast, and malt medium (SYM), A3M, and BPM (recipes below) (500 mL in 2 L baffled flasks) was inoculated at 1% final culture volume from the overnight cultures and grown by shaking at 28°C and 220 rpm for 8 days. The supernatant was harvested by centrifugation at 10,000 *g* for 5 minutes and concentrated 20× from its original fermentation via rotary evaporator or lyophilization. Two microliters of each concentrated extract was spotted onto Mueller-Hinton II agar (MHIIA) plates overlayed with a lawn of bacteria (*Escherichia coli, Pseudomonas aeruginosa,* and *Staphylococcus aureus*) back diluted to 0.03 OD from a 3 mL overnight culture in Mueller-Hinton II broth (MHIIB). Plates were allowed to grow stationary and inverted at 37°C for 16 hours, after which they were observed for zones of inhibition. SYM was selected for use in further screening as it was the condition that allowed for the greatest amount of antimicrobial activity.

### Media

All per 1 L water. SYM: 4 g soluble starch (Acros Organics), 4 g yeast extract (BD), and 8 g malt (BD). R4: 10 g glucose (Alfa Aesar), 1 g yeast extract (BD), 0.1 g casamino acids (OmniPur EMD), 3 g proline (Alfa Aesar), 10 g magnesium chloride hexahydrate (Fisher Scientific), 4 g calcium chloride dihydrate (Fisher Scientific), 0.2 g potassium sulfate (EMD), 5.6 g TES (Sigma-Aldrich), 1 mL trace elements, and 0.45 mL 10 M KOH or NaOH. A3M: 5 g glucose (Alfa Aesar), 20 mL glycerol (Acros Organics), 20 g soluble starch (Acros Organics), 15 g Pharmamedia (Traders Protein), 3 g yeast extract (BD), and 10 g diaion HP-20. BPM: 20 g glucose (Alfa Aesar), 1 g ammonium sulfate (Acros Organics), 20 g glycerol (Acros Organics), 10 g Pharmamedia (Traders Protein), 10 g calcium carbonate (Acros Organics), and 10 g soy flour (Red Mill, organic).

### Isolation and mass determination of DHB

*P. laumondii* T181 (GenBank: JBEMBR000000000) was streaked from a cryostock to a plate of TSA, then inoculated into a 5 mL TSB overnight culture shaking at 28°C and 220 rpm. A starter culture was used to inoculate 500 mL of SYM pH 7.2 in 2 L baffled flasks at 1% final culture volume and grown in a 28°C shaking incubator (220 rpm) for 8 days. *P. laumondii* supernatant was then harvested by centrifugation (10,000 *g* for 5 minutes) and purified using anion exchange chromatography. The supernatant containing the active compound was loaded onto a column containing 180 mL of Q-FF resin. Supernatant pH was adjusted to 10 using 1 M NaOH before loading. A stepwise elution gradient was utilized at pH 10, 9, 7, 5, and 3, using approximately 10 column volumes (2 L) of 50 mM ammonium acetate for each step. Active compound eluted at the pH 3 step was concentrated 1,000× relative to supernatant on a rotary evaporator followed by EZ-2 Plus GeneVac for further isolation. The concentrated extract was injected onto a reversed-phase high-performance liquid chromatography (RP-HPLC) instrument (Agilent 1260 HPLC system, Agilent Technologies) using a C18 semi-preparative scale column (XBridge, 250 × 10 mm, 5 µm, 130 Å, Waters). RP-HPLC conditions were as follows: solvent A, ddH_2_O with 0.1% (vol/vol) formic acid; solvent B, acetonitrile with 0.1% (vol/vol) formic acid. A flow rate of 3 mL/minute and a gradient of 2%–95% solvent B was used over 20 minutes. A photodiode array enables UV monitoring for peak picking. Twelve major peaks were observed and collected by hand for activity testing (Fig. S1) Pure compound was simultaneously tested for consistent activity against wild-type *E. coli* and injected on the LC-MS (Agilent 6530 quadrupole time-of-flight with electrospray ionization, coupled to Agilent 1260 HPLC system, Agilent Technologies). MS parameters were as follows: collision gas, ultra-high purity N_2_; gas temperature, 300°C; gas flow, 7 L/minute; nebulizer, 35 psi; fragmentor voltage, 175 V; and skimmer voltage, 65 V. Acquisition was set in positive mode at “Auto MS/MS” with the following parameters: mass range for MS 111–3,000 *m/z* with 2 spectra/s; mass range for MS/MS, 50–3,000 *m/z* with 4 spectra/s; max precursor per cycle, 10; active exclusion after three spectra with release after 0.5 minutes. MassHunter software (Agilent Technologies) was used for data acquisition and qualitative analysis (Fig. S2). The ^1^H NMR spectrum was measured on a Bruker Avance Neo 500 MHz NMR spectrometer with a broadband BBFO probe. The experiment was conducted with 1 mg of compound solubilized in 600 µL of CD_3_OD. Chemical shifts were referenced to the respective residual solvent peak (δ_H_ 3.31). Preliminary structure was determined with the use of ^1^H NMR spectrum 7.50 (d, *J* = 8.6 Hz, ^1^H), 6.74 (dd, *J* = 8.6, 1.9 Hz, ^1^H), and 6.67 (d, *J* = 1.9 Hz, ^1^H) (Fig. S3). The resulting ^1^H NMR spectrum of the compound was in agreement with that of 3,6-dihydroxy-1,2-benzisoxazole in the literature (21, 22). After the initial isolation of DHB from *P. laumondii*, this compound as well as ^15^N-labeled DHB was purchased from Life Chemicals, Inc. for further testing.

### BGC identification

Adapting the protocol from reference (22), 1 µL of *Vibrio* isolate (GenBank: JBELYB000000000) (previously annotated as *Bradyrhizobium denitrificans*) from Drs. Kristen Whalen and David Rowley was inoculated into a 3 mL MB2216 seed culture, which was grown for 16 hours at 28°C. The seed culture was back diluted 0.1% into 1 L Marine Broth 2216 (MB2216) (Difco) at 0.1% of the final culture volume in a 2.8 L baffled flask (Kontes), which was grown at 28°C shaking at 190 rpm for 8 days. The supernatant was collected by centrifugation, filtered, and then added to a 20 mL 1:1 mixture of washed Amberlite XAD-7 and XAD-16N resins. The mixture was co-incubated by shaking at room temperature for 16 hours, after which resin was collected by filtration and eluted by shaking in 1 L of methanol for 3 hours. The resin was again removed by filtration, and methanol was removed by rotary evaporator. The resulting dried crude extract was analyzed on the LC-MS to confirm the presence of DHB. Other *Photorhabdus* producers, including environmental isolate *P. laumondii* S56 (GenBank: JBELYA000000000), were grown initially from cryostock as previously described for *P. laumondii* T181, then inoculated in 1 L TSB in 2 L baffled flasks at 1% final culture volume and grown in a 28°C shaking incubator (220 rpm) for 8 days. The supernatant was harvested via centrifugation (10,000 *g* for 5 minutes), purified using XAD-16N resin, and analyzed on the LC-MS as described above.

Every gene in *P. laumondii* TT01 was searched using NCBI tBLASTn against the nucleotide genomes of each other strain listed, using an *E*-value cutoff of 10^−20^ and an identity cutoff of 70% for *Photorhabdus* species or an *E*-value cutoff of 10^−10^ and an identity cutoff of 50% for comparisons to the *Vibrio* isolate. Genes with hits passing these thresholds were considered to be present in the other species, while those below these cutoffs were considered to be absent. A list was then compiled of all genes that were only present in the DHB producers and absent in all non-producing strains. These “producer-only” genes and their immediate surroundings on the *P. laumondii* TT01 genome were then evaluated to see if they could plausibly represent a BGC for DHB, using their annotations and the top hits from a UniProtKB BLAST search to determine possible functions for each gene. This included both BGCs composed primarily of producer-only genes as well as cases where as few as one producer-only gene was present but could be a required factor for compound production in its surrounding putative BGC. Considering the possibility that one of the “non-producer” strains might possess the BGC but produce DHB only at levels below our limit of detection, the above analysis was also performed on all cases where at least two “rare” genes, present in the three producer strains but also at most one non-producer strain, were co-clustered. This analysis yielded only one candidate BGC for the biosynthesis of DHB. The boundaries of the BGC were determined by examining which genes were present on both *P. laumondii* and *Vibrio* versions of the cluster.

Homologous BGCs in other species were identified using cblaster (51). The phylogeny of the BGCs from selected strains was determined from a multiple sequence alignment of the nucleotide sequence of the full BGCs, performed using Clustal Omega. Trees were visualized with the help of the Interactive Tree of Life tool (52).

### MIC and cytotoxicity testing

Pathogenic strains were grown in 3 mL of Mueller-Hinton II broth (MHIIB) and diluted to OD_600_ = 0.001. A volume of 100 µL of diluted bacterial culture was added in duplicate to a 96-well round bottom plate containing a twofold serial dilution of DHB with the highest concentrations tested at 128 µg/mL. Plates were incubated for 16 hours, and OD_600_ was measured with a Synergy H1 microplate reader. Growth inhibition of 70% or greater compared to the untreated control indicated the minimum inhibitory concentration. Cytotoxicity assays were performed in triplicate using a microplate Alamar blue assay against HepG2, FaDu, and HEK293 human cell lines. DHB was twofold serially diluted in Eagle’s minimum essential media in a 96-well plate and added to human cells after allowing growth to establish for 24 hours. After 72 hours, the redox-sensitive dye resazurin is added to each well and incubated for 3 hours. Absorbances were read at 544 and 590 nm on a microplate reader. All graphical representations of MIC data show biological replicates and standard deviations (SD).

Anaerobic strains were initially revived in Hungate anaerobic culture tubes for 3 days in Gifu Anaerobic Broth (GAM) containing 0.5 g/L of cysteine and 0.0001% resazurin. Strains were passaged 1:100 into fresh anaerobic Hungate tubes 24 hours before the MIC test. In an anaerobic chamber, the strains were diluted 1:100 in GAM containing 0.5 g/L cysteine and 0.0001% resazurin. DHB was dissolved in aerobic DMSO for a final concentration of 128 µg/mL. The drug was diluted 1:1 until the concentration reached 0.0625 µg/mL in a final volume of 100 µL per well. MICs were performed in duplicate and included media only, medium with drug, and medium with cells-only controls. Plates were sealed to prevent evaporation and incubated anaerobically at 37°C. Plates were read visually 24 hours later. The presence of oxidized resazurin in the media prevents the use of the plate reader. Strains were sequenced to confirm taxonomy.

### Selection for resistant mutants

Single colonies of *E. coli* MG1655 were picked from a MHIIA plate and inoculated into 3 mL MHIIB overnight cultures incubated shaking at 220 rpm and 37°C. The next day, culture was added at 0.1% of final volume to 250 mL flasks containing 50 mL MHIIB media and grown at 37°C shaking at 220 rpm to an OD_600_ of 0.5. One milliliter of 5 × 10^9^, 5 × 10^7^, and 5 × 10^5^ cells were plated on MHIIA plates containing 4× MIC (4 µg/mL) and 16× MIC (16 µg/mL) of DHB. Colonies began to appear on 5 × 10^9^ plates after 3 days of static incubation at 37°C and were robust enough to harvest after 5–7 days. After 12 days, all plates but 16× MIC 5 × 10^7^ and 5 × 10^5^ contained colonies. Colonies were passaged onto 4× MIC DHB plates to ensure resistance and tested for an increased MIC. Mutants with the highest MICs were sent to SeqCenter for Illumina sequencing and variant calling. Sample libraries were prepared using the Illumina DNA Prep kit and IDT 10 bp UDI indices and sequenced on an Illumina NextSeq 2000, producing 2 × 151 bp reads. Demultiplexing, quality control, and adapter trimming were performed with bcl-convert (version 3.9.3). Variant caller breseq (version 0.36.1) was used to align and compare sequencing data to a reference *E. coli* MG1655 file (47). For sequencing of *ΔubiA* mutants, the four most robust colonies were picked from an MHIIA plate incubated aerobically at 37°C. Colonies were inoculated into 3 mL overnight cultures and grown by shaking at 220 rpm and 37°C. Cell pellets were sent for full genome sequencing and variant calling as previously described.

### Molecular modeling

Maestro (version 13.7.125) by Schrödinger was used to investigate DHB’s binding profile to UbiA. Briefly, *ab initio E. coli* protein models were generated using AlphaFold2, minimized with amber, and aligned to the previously published ligand-free and bound models from *Aeropyrum pernix* K1 (PDB:4OD4 and 4OD5, respectively). The natural substrates were transplanted into the active site of the *ab initio E. coli* model via superposition, with an RMSD of 4.6 Å^3^ (reference PDB: 4OD5) (53). Following this, the geranyl-S-thiolodiphosphate was manually edited to geranyl-s-pyrophosphate. This structure was minimized (OPLS-20) using Protein Preparation Workflow, which assigned bond orders, added hydrogens, created zero-order bonds to metals, generated disulfide bonds, filled in missing side chains and loops using Prime, generated het states using Epik at pH 7.0 ± 2.0, and deleted water molecules beyond 5 Å from het groups (54). The resultant system was used for docking studies using the Glide workflow, the grid was defined around 4-HBA. DHB and 4-HBA underwent ligand preparation using LigPrep to create energy-minimized three-dimensional structures. The OPLS3e force field was used for minimization. Epik was used to generate all the possible ionized states at pH 7.0 ± 2.0. The desalt setting was used to remove any counter ions or water molecules. Tautomers and stereoisomers were generated (at most 32 per ligand) where specified chiralities were retained. 4HBA and DHB were then docked at XP (extra-precision). The 4-HBA was found to resemble the crystal structure pose with an RMSD of 0.15 Å^3^ validating the docking grid. The XP docking results found that DHB binds modestly better to UbiA (−7.1 v. −6.3 kcal/mol). This suggested that DHB has an improved Gibbs Free energy profile in the binding domain of UbiA. To assess this further, molecular dynamic simulations were performed in Desmond on an Nvidia RTX 4070ti. Here, the poses from the XP docking were used as starting geometries. In an orthorhombic 10 Å^3^ unit cell, a membrane was established with DPPC, with domains set to the helices using TMPDB (55). The TIP3P solvent system was used, and each system was neutralized using automatic ion placement. Simulations were performed for 100 ns (recording every 50 ps, ensemble class NPT, temperature 300 K, and 1.01325 bar). Following simulation, the 2,000-frame trajectories were reviewed for 4-HBA and DHBs within ~10 ns of simulation, indicating equilibrium by a stable radius of gyration (rG) ranging from 20 to 24 Å for both models, which is in line with previous literature (45). As such, the equilibrium frames (200–2,000) were used for molecular mechanics with generalized Born and surface area solvation (mm/gbsa) calculations of the ligand in the active site. This followed the lower accuracy docking results showing ADC to bind at −30.5 (2) vs −24.1 (5) kcal/mol in UbiA.

### Cloning

#### Overexpression constructs

To make overexpression strains *pmmB67EH::ubiA, pmmB67EH::ubiC,* and *pmmB67EH::aaeB,* the gene of interest was amplified from *E. coli* MG1655 using primers with homology to both *ubiA* and plasmid *pmmB67EH*. Primers are as follows: *ubiA* F (5′ agaattcgagctcggtacccattaaagaggagaaattaactatgGAGTGGAGTCTGACGCA 3′), R (5′ gtcgactctagaggatcccctcaGAAATGCCAGTAACTCATTGC 3′); *ubiC* F (5′ agaattcgagctcggtacccattaaagaggagaaattaactatgTCACACCCCGCGTTAAC 3′), R (5′ gtcgactctagaggatccccttaGTACAACGGTGACGCCG 3′); and *aaeB* F (5′ agaattcgagctcggtacccattaaagaggagaaattaactatgGGTATTTTCTCCATTGC 3′), R (5′ gtcgactctagaggatccccttaACTATCGGTCAACGCAT 3′). Inserts were annealed to the plasmid via Gibson Assembly (NEB Gibson Assembly Master Mix, 1 hour at 50°C). Gibson products (plasmids with inserts and plasmids only as a negative control) were transformed into CaCl_2_-competent *E. coli* MG1655 cells as described below, plated on LBA with 50 mg/mL carbenicillin, and incubated overnight at 37°C. All plasmids were confirmed via sequencing.

#### Chemically competent cells

A single colony of bacteria intended for chemical competency was inoculated into 5 mL LB and grown overnight shaking at 220 rpm at 37°C. The following day, cells were inoculated at 1% of final volume into LB and grown in a 37°C shaking incubator at 220 rpm until they reached an OD_600_ of 0.375. Cultures were aliquoted into 50 mL prechilled sterile polypropylene tubes and kept on ice for 10 minutes. Cells were then centrifuged for 7 minutes at 1,600 × *g* at 4°C. The lowest brake setting was used on the centrifuge. The supernatant was decanted off, and each pellet was gently resuspended in 10 mL ice-cold CaCl_2_ solution (60 mM CaCl_2_, 15% glycerol, and 10 mM PIPES pH 7, filter sterilized). Cells were centrifuged for 5 minutes at 1,100 × *g* at 4°C, the liquid was decanted off, and the resulting pellets were again resuspended in 10 mL ice-cold CaCl_2_ solution. This mixture was kept on ice for 30 minutes, then centrifuged again for 5 minutes at 1,100 × *g* at 4°C. The supernatant was poured off, and each pellet was resuspended in 2 mL ice-cold CaCl_2_ solution. Cells were dispensed into prechilled microcentrifuge tubes (250 µL aliquots) and immediately placed into a −80°C freezer.

#### Transformations

Chemically competent cells were thawed on ice, then added in 50 µL aliquots with 1–5 μL of plasmid to each transformation tube. Tubes were kept on ice for 15 minutes, then heat shocked at 42°C for 90 s. Tubes were placed back on ice for 3 minutes, after which 1 mL of prewarmed LB was added to each tube. Cells were recovered for 1 hour at 37°C shaking at 220 rpm. A volume of 100 µL was spread over LBA plates with antibiotic. The remaining 900 µL of cells was centrifuged at 8,000 × *g* for 3 minutes before 800 µL of the supernatant was removed. The cell pellet was resuspended in the remaining 100 µL of LB and spread on LBA plates containing antibiotic.

### Knockout strains

#### Lambda Red-mediated recombination

Strains *ΔubiAC, ΔtolC,* and *ΔhemB* were made using Lambda Red-mediated recombination. To make electrocompetent cells, *E. coli* MG1655 carrying lambda (λ) plasmid pKD46 was grown overnight shaking at 220 rpm in 3 mL LB with 50 mg/mL carbenicillin at 30°C. The overnight culture was diluted 100-fold into growth media consisting of 2 M MgCl_2_ and 200 mM L-arabinose in LB carbenicillin and grown at 30°C shaking at 220 rpm for 3 hours. Upon reaching an OD_600_ of 0.4–0.6, cells were spun at 9,000 rpm for 5 minutes, and the pellet was washed two times with dH_2_O. One to five microliters of Kanamycin cassette PCR amplified from vector pKD4 (F 5′ TAACAGAACTGTTTTTACCGGCGTCACCGTTGTACTAAGAGGAAAAAAATTGTAGGCTGGAGCTGCTTCG 3′, R 5′ ATCAGGCAACCCAGAAGAAAGCCGGATGATCATCCGGCTTTTTTACATCAATGGGAATTAGCCATGGTCC 3′) was added to 50 µL of electrocompetent cells. Each reaction mix was transferred to an electroporation cuvette (1 mm) and electroporated at 1.25 kV. Cells were immediately recovered with 1 mL of prewarmed SOC medium (SOB medium 20 g tryptone, 5 g yeast extract, and 0.5 g NaCl added to 1 L dH_2_O and autoclaved, followed by the addition of 10 mL 1 M MgCl_2_, 10 mL 1 M MgSO_4_, and 2 mL 20% glucose to SOB for a final volume of 100 mL SOC medium). Transformants were recovered by shaking at 37°C for 3 hours, plated on prewarmed LBA plates with 50 mg/mL kanamycin, and allowed to grow overnight at 37°C. *ΔubiAC* and *ΔhemB* LBA plates were supplemented with 0.4% glucose. The following day, recombinant cells were picked and checked for the presence of the kanamycin cassette via gel electrophoresis as well as Sanger sequencing following PCR amplification of the target gene. *ΔubiA* was gifted by Dr. Fabien Pierrel at Grenoble Alpes University after Lambda Red-mediated recombination under anaerobic conditions. Full genome sequencing and variant calling performed on *ΔubiA* showed that colonies able to grow aerobically had single point mutations in NADH:quinone oxidoreductase subunits M, N, or G (*nuoM, nuoN,* or *nuoG*) (Table S6). These enzymes make up part of a complex that catalyzes NADH oxidation coupled with ubiquinone reduction to transport protons across the inner membrane. Mutations in individual *nuo* genes cause changes in the activity of the complex (56).

#### P1 phage transduction

Strains *ΔubiC* and *ΔaaeB* were transduced via P1 phage into an *E. coli* MG1655 background from corresponding knockouts in the KEIO collection (57). To make the phage, 100 µL of lambda P1 phage stock and 100 µL of donor culture (from KEIO collection, single colony inoculated into 3 mL LB and grown overnight shaking at 220 rpm and 37°C) were added to LB media with 7 mM CaCl_2_ and 12 mM MgSO_4_. The mixture was incubated stationary at 37°C for 30 minutes to allow for phage attachment. This was followed by 2 hours of shaking at 220 rpm and 37°C. A volume of 1 mL of chloroform was added to the phage mixture, and the sample was vortexed for 2 minutes, then centrifuged at 12,100 rpm for 5 minutes at room temperature. The upper layer was then collected in a new tube and a drop of chloroform was added.

Using the phage made from the donor cells, we prepared three tubes for the transduction. To separate tubes containing 1 mL of LB with 7 mM CaCl_2_ and 12 mM MgSO_4_, 400 and 40 µL of P1 phage were added. The third tube contained media only (no P1 phage). Recipient cells were inoculated from a single colony the night before into 3 mL LB shaking at 220 rpm and 37°C. A volume of 150 µL of the recipient culture was added to each tube. The resulting mixtures were incubated stationary at 37°C for 25 minutes, then centrifuged at 14,000 rpm for 5 minutes at room temperature. The supernatant was discarded, and the pellet was resuspended in 200 µL of LB with 25 mM citrate. Transductions were spread onto LBA plates containing 25 mM citrate and 50 mg/mL kanamycin and incubated overnight at 37°C. All resulting colonies were checked for the presence of the kanamycin cassette as described above.

#### *ubiA* swap

*ubiA* from *P. aeruginosa* PAO1 was codon optimized for *E. coli* expression and synthesized via IDT. After amplification (F 5′ agaattcgagctcggtacccattaaagaggagaaattaactATGTTCGTTACCCTTATTAAACCC 3′, R 5′ gtcgactctagaggatccccTCAGCGAAGTGCATAATCCG 3′), PAO1 *ubiA* was inserted into plasmid *pmmB67EH* as described above. *pmmB67EH::ubiA* PAO1 was transformed into CaCl_2_ competent *ΔubiA* cells as described above and sequenced for confirmation. MDR pump knockout strain *P. aeruginosa* PAO1 PΔ6 and PAO1-Pore were given by Dr. Helen I. Zgurskaya of the University of Oklahoma.

### Metabolomics

Triplicate 3 mL cultures of *E. coli* MG1655 (wild type), *pmmB67EH::ubiA* (overexpression)*,* and *ΔubiA* (knockout) from three independent colonies per strain were grown shaking overnight at 37°C, 220 rpm and inoculated 1:10,000 into 50 mL MHIIB in 125 mL flasks the next day. Overexpression construct *pmmB67EH::ubiA* was grown to OD_600_ 0.1–0.15 and induced with 50 µL of 1 M isopropyl β-D-1-thiogalactopyranoside (final concentration 1 mM). After all cultures reached OD_600_ 0.2–0.3, cultures were split into two 25 mL cultures in separate 125 mL flasks and half were treated with 10× MIC DHB (10 µg/mL). Treated and untreated cultures were allowed to grow by shaking at 37°C and 220 rpm for 1 hour. Final OD_600_ was recorded, and cells were spun down for 5 minutes at 10,000 × *g* at 4°C. Pellets were resuspended in 5 mL 100% methanol per 25 mL culture (10 mL methanol to MG1655 wild-type cultures to keep biomass the same). Cells were lysed via sonication (Heat systems—Ultrasonics, Inc., model W185) for 1 minute at power 3 and spun for 20 minutes at 14,000 *× g*. An internal standard of ring-^13^C6-labeled 4-hydroxybenzoate (Cambridge Isotope Laboratories) was added for a final concentration of 20 nM. A volume of 100 µL of 100 µg/mL 4-hydroxybenzoic acid, chorismic acid, and DHB were sent as reference standards, along with the structure and mass of 4-hydroxy-3-octaprenylbenzoate and octaprenyl pyrophosphate. A volume of 5 mL of each sample was sent to the Harvard Center for Mass Spectrometry. All samples were dried under N_2_, resuspended in 30 µL of 50% acetonitrile in water, and run on a ThermoFisher ID-X mass spectrometer (Zic pHILIC column 150 × 2.1 mm 5 µm). LC method was as follows: 5 µL injection volume with 0–0.5 minutes 93% solvent B (acetonitrile 97% in water), with solvent A (20 mM ammonium carbonate, 0.1% ammonium hydroxide in water) increased linearly from 0.5 to 28 minutes. Solvent A remains at 100% from 28 to 33 minutes and drops back to 7% from 36 to 45 minutes. Flow is increased from an initial 0.05 mL/minute (0–0.5 minutes) to 0.15 mL/minute. Ratios of peak areas divided by the internal standard peak (^13^C6 4-HB) were calculated for all targeted reference standards. Peak extraction, retention time alignment, gap filling, background subtraction, normalization, and compound identity determination were all performed by compound discoverer version 3.3. Adjusted *P*-values were calculated using the ordinary one-way ANOVA method followed by a Tukey’s *post hoc* test as implemented in GraphPad Prism.

Overnight cultures of *pmmB67EH::ubiA* were back diluted, grown, and induced as described above. Cultures were treated with either 10× MIC unlabeled ^14^N DHB, labeled ^15^N DHB, or a 1:1 ratio of ^14^N:^15^N DHB. Untreated culture was included as a control. Cells were lysed and sent to Harvard Center of Mass Spectrometry, where they were dried down under N_2_ flow and resuspended in 30 µL of 50% methanol in water. All samples were run on a ThermoFisher ID-X mass spectrometer on a Kinetex EVO C18 150 × 2.1 mm column at 40°C. A volume of 5 µL of each sample held at 4°C was injected and run with the following method: 0–5 minutes of 0% acetonitrile with 0.1% formic acid (B), 5–15 minutes ramp from 0% to 100% B, 15–35 minutes hold at 100% B, and 35.05–40 minutes 0% B. A labeling check was performed by comparing the predicted and observed mass spectra of ^14^N and ^15^N DHB standards. Standards were shown to be labeled as expected and with total separation between ^14^N and ^15^N DHB at the resolution of the instrument. SIM on 698.5507, 700.5299, 714.5456, and 728.5612 was performed with 3 *m/z* isolation. The mass of prenylated DHB (DHB_8_) was accurate for the predicted formula at −0.2 ppm with anything below 5 ppm considered to be highly accurate.

### Membrane potential

3,3′-Diethyloxacarbocyanine iodide [DiOC_2_(3)] is a positively charged green fluorescent dye that accumulates in cells with increasing membrane potential, forming aggregates and shifting the fluorescence spectrum from red to green (58). Single colonies of *E. coli* MG1655 were picked from an MHIIA plate and inoculated into 5 mL MHIIB overnight cultures shaking at 220 rpm and 37°C. After a 16-hour incubation, cells were added to 5 mL fresh MHIIB at 1% of the final volume. A 5 mL culture was split into two 2.5 mL cultures when cells reached an OD_600_ of 0.2–0.3. One culture was treated with 32× MIC DHB, and the other was left as an untreated control. Cells were added at 1% (exponential) or 0.5% of final volume (stationary) at 0, 30, 60, and 90 minutes to 1 mL prewarmed PBS containing 0.1 mM EDTA and 30 µM DiOC_2_(3) and incubated for 10 minutes at 37°C. DHB-treated stationary culture was added at 1% of the final volume to adjust for growth suppression. Cells were also added to a no DiOC_2_(3) control and a carbonyl cyanide m-chlorophenyl hydrazone (CCCP) positive control, which contained the standard PBS, EDTA, and DiOC_2_(3) mixture with an added 1 µM CCCP. CCCP is a protonophore that disrupts membrane potential by transporting protons across the inner membrane. Cells were immediately analyzed on a fluorescence-activated cell sorting (BD FACS Aria II) machine for green fluorescence (FITC channel) and red fluorescence (mCherry channel). The ratio of red/green channel emission was used to measure proton motive force.

## Data Availability

Full metabolomic data are available in MetaboLights under identifier MTBLS10720. GenBank accession numbers are as follows: for *Photorhabdus laumondii* T181, JBEMBR000000000; for *Photorhabdus laumondii* S56, JBELYA000000000; and for *Vibrio neptunius* B158, JBELYB000000000.

## SUPPLEMENTAL MATERIAL

Supplemental material mbio.01167-24-s0001.docx Figures S1 to S9; Tables S1 to S6.

ASM does not own the copyrights to Supplemental Material that may be linked to, or accessed through, an article. The authors have granted ASM a non-exclusive, world-wide license to publish the Supplemental Material files. Please contact the corresponding author directly for reuse.

## References

[1] Zgurskaya HI, Löpez CA, Gnanakaran S. 2015. Permeability barrier of Gram-negative cell envelopes and approaches to bypass it. ACS Infect Dis 1:512–522. doi:10.1021/acsinfecdis.5b00097 26925460 PMC4764994

[2] Lesher GY, Froelich EJ, Gruett MD, Bailey JH, Brundage RP. 1962. 1,8-Naphthyridine derivatives. A new class of chemotherapeutic agents. J Med Chem 5:1063–1065. doi:10.1021/jm01240a021 14056431

[3] Emmerson AM, Jones AM. 2003. The quinolones: decades of development and use. J Antimicrob Chemother 51 Suppl 1:13–20. doi:10.1093/jac/dkg208 12702699

[4] Rice LB. 2008. Federal funding for the study of antimicrobial resistance in nosocomial pathogens: no ESKAPE. J Infect Dis 197:1079–1081. doi:10.1086/533452 18419525

[5] Aminov RI. 2010. A brief history of the antibiotic era: lessons learned and challenges for the future. Front Microbiol 1:134. doi:10.3389/fmicb.2010.00134 21687759 PMC3109405

[6] Lewis K. 2020. The science of antibiotic discovery. Cell 181:29–45. doi:10.1016/j.cell.2020.02.056 32197064

[7] Tobias NJ, Shi Y-M, Bode HB. 2018. Refining the natural product repertoire in entomopathogenic bacteria. Trends Microbiol 26:833–840. doi:10.1016/j.tim.2018.04.007 29801772

[8] Crawford JM, Clardy J. 2011. Bacterial symbionts and natural products. Chem Commun (Camb) 47:7559–7566. doi:10.1039/c1cc11574j 21594283 PMC3174269

[9] Tambong JT. 2013. Phylogeny of bacteria isolated from Rhabditis sp. (Nematoda) and identification of novel entomopathogenic Serratia marcescens strains. Curr Microbiol 66:138–144. doi:10.1007/s00284-012-0250-0 23079959

[10] Imai Y, Meyer KJ, Iinishi A, Favre-Godal Q, Green R, Manuse S, Caboni M, Mori M, Niles S, Ghiglieri M, et al.. 2019. A new antibiotic selectively kills Gram-negative pathogens. Nature 576:459–464. doi:10.1038/s41586-019-1791-1 31747680 PMC7188312

[11] Miller RD, Iinishi A, Modaresi SM, Yoo B-K, Curtis TD, Lariviere PJ, Liang L, Son S, Nicolau S, Bargabos R, Morrissette M, Gates MF, Pitt N, Jakob RP, Rath P, Maier T, Malyutin AG, Kaiser JT, Niles S, Karavas B, Ghiglieri M, Bowman SEJ, Rees DC, Hiller S, Lewis K. 2022. Computational identification of a systemic antibiotic for Gram-negative bacteria. Nat Microbiol 7:1661–1672. doi:10.1038/s41564-022-01227-4 36163500 PMC10155127

[12] Shahsavari N, Wang B, Imai Y, Mori M, Son S, Liang L, Böhringer N, Manuse S, Gates MF, Morrissette M, Corsetti R, Espinoza JL, Dupont CL, Laub MT, Lewis K. 2022. A silent operon of Photorhabdus luminescens encodes a prodrug mimic of GTP. mBio 13:e0070022. doi:10.1128/mbio.00700-22 35575547 PMC9239236

[13] Pantel L, Florin T, Dobosz-Bartoszek M, Racine E, Sarciaux M, Serri M, Houard J, Campagne J-M, de Figueiredo RM, Midrier C, Gaudriault S, Givaudan A, Lanois A, Forst S, Aumelas A, Cotteaux-Lautard C, Bolla J-M, Vingsbo Lundberg C, Huseby DL, Hughes D, Villain-Guillot P, Mankin AS, Polikanov YS, Gualtieri M. 2018. Odilorhabdins, antibacterial agents that cause miscoding by binding at a new ribosomal site. Mol Cell 70:83–94. doi:10.1016/j.molcel.2018.03.001 29625040

[14] Modi SR, Collins JJ, Relman DA. 2014. Antibiotics and the gut microbiota. J Clin Invest 124:4212–4218. doi:10.1172/JCI72333 25271726 PMC4191029

[15] Willing BP, Russell SL, Finlay BB. 2011. Shifting the balance: antibiotic effects on host-microbiota mutualism. Nat Rev Microbiol 9:233–243. doi:10.1038/nrmicro2536 21358670

[16] Schuster M, Brabet E, Oi KK, Desjonquères N, Moehle K, Le Poupon K, Hell S, Gable S, Rithié V, Dillinger S, Zbinden P, Luther A, Li C, Stiegeler S, D’Arco C, Locher H, Remus T, DiMaio S, Motta P, Wach A, Jung F, Upert G, Obrecht D, Benghezal M, Zerbe O. 2023. Peptidomimetic antibiotics disrupt the lipopolysaccharide transport bridge of drug-resistant Enterobacteriaceae. Sci Adv 9:eadg3683. doi:10.1126/sciadv.adg3683 37224246 PMC10208570

[17] Muñoz KA, Ulrich RJ, Vasan AK, Sinclair M, Wen P-C, Holmes JR, Lee HY, Hung C-C, Fields CJ, Tajkhorshid E, Lau GW, Hergenrother PJ. 2024. A Gram-negative-selective antibiotic that spares the gut microbiome. Nature 630:429–436. doi:10.1038/s41586-024-07502-0 38811738 PMC12135874

[18] Zampaloni C, Mattei P, Bleicher K, Winther L, Thäte C, Bucher C, Adam J-M, Alanine A, Amrein KE, Baidin V, et al.. 2024. A novel antibiotic class targeting the lipopolysaccharide transporter. Nature 625:566–571. doi:10.1038/s41586-023-06873-0 38172634 PMC10794144

[19] Leimer N, Wu X, Imai Y, Morrissette M, Pitt N, Favre-Godal Q, Iinishi A, Jain S, Caboni M, Leus IV, et al.. 2021. A selective antibiotic for Lyme disease. Cell 184:5405–5418. doi:10.1016/j.cell.2021.09.011 34619078 PMC8526400

[20] Schubert AM, Sinani H, Schloss PD. 2015. Antibiotic-induced alterations of the murine gut microbiota and subsequent effects on colonization resistance against Clostridium difficile. mBio 6:e00974. doi:10.1128/mBio.00974-15 26173701 PMC4502226

[21] Imai H, Suzuki K-I, Miyazaki S, Tanaka K, Watanabe S, Iwanami M. 1983. A new antibiotic Y-T0678H produced by a Chromobacterium species. J Antibiot 36:911–912. doi:10.7164/antibiotics.36.911 6615590

[22] Deering RW, Whalen KE, Alvarez I, Daffinee K, Beganovic M, LaPlante KL, Kishore S, Zhao S, Cezairliyan B, Yu S, Rosario M, Mincer TJ, Rowley DC. 2021. Identification of a bacteria-produced benzisoxazole with antibiotic activity against multi-drug resistant Acinetobacter baumannii. J Antibiot (Tokyo) 74:370–380. doi:10.1038/s41429-021-00412-7 33580212 PMC7879144

[23] Pelosi L. 2019. Ubiquinone biosynthesis over the entire O_2_ range: characterization of a conserved O_2_-independent pathway. mBio 10. doi:10.1128/mBio.01319-19 PMC6747719 31289180

[24] Søballe B, Poole RK. 1999. Microbial ubiquinones: multiple roles in respiration, gene regulation and oxidative stress management. Microbiology (Reading) 145 ( Pt 8):1817–1830. doi:10.1099/13500872-145-8-1817 10463148

[25] Nowicka B, Kruk J. 2010. Occurrence, biosynthesis and function of isoprenoid quinones. Biochim Biophys Acta 1797:1587–1605. doi:10.1016/j.bbabio.2010.06.007 20599680

[26] Collins MD, Jones D. 1981. Distribution of isoprenoid quinone structural types in bacteria and their taxonomic implication. Microbiol Rev 45:316–354. doi:10.1128/mr.45.2.316-354.1981 7022156 PMC281511

[27] COX GB, GIBSON F. 1964. Biosynthesis of vitamin K and ubiquinone relation to the shikimic acid pathway in Escherichia coli. Biochim Biophys Acta 93:204–206. doi:10.1016/0304-4165(64)90285-5 14249157

[28] Olson RE, Rudney H. 1983. Biosynthesis of ubiquinone, p 1–43. In Aurbach GD, McCormick DB (ed), Vitamins & hormones. Vol. 40. Academic Press. 10.1016/s0083-6729(08)60431-8 6369767

[29] Goewert RR. 1980. Studies on the biosynthesis of ubiquinone: the identification of 3,4-dihydroxy-5-hexaprenylbenzoic acid, 3-methoxy-4-hydroxy-5-hexaprenylbenzoic acid and the regulation of the conversion of tyrosine and chorismic acid to ubiquinone in Saccharomyces cerevisiae. Missouri, United States Saint Louis University

[30] Aussel L, Pierrel F, Loiseau L, Lombard M, Fontecave M, Barras F. 2014. Biosynthesis and physiology of coenzyme Q in bacteria. Biochim Biophys Acta 1837:1004–1011. doi:10.1016/j.bbabio.2014.01.015 24480387

[31] Somvanshi VS, Dubay B, Kushwah J, Ramamoorthy S, Vishnu US, Sankarasubramanian J, Rajendhran J, Rao U. 2019. Draft genome sequences for five Photorhabdus bacterial symbionts of entomopathogenic Heterorhabditis nematodes isolated from India. Microbiol Resour Announc 8:10. doi:10.1128/MRA.01404-18 PMC6346184 30701235

[32] Van Dyk TK, Templeton LJ, Cantera KA, Sharpe PL, Sariaslani FS. 2004. Characterization of the Escherichia coli AaeAB efflux pump: a metabolic relief valve? J Bacteriol 186:7196–7204. doi:10.1128/JB.186.21.7196-7204.2004 15489430 PMC523213

[33] Barker JL, Frost JW. 2001. Microbial synthesis of p-hydroxybenzoic acid from glucose. Biotechnol Bioeng 76:376–390. doi:10.1002/bit.10160 11745165

[34] Zaldivar J, Ingram LO. 1999. Effect of organic acids on the growth and fermentation of ethanologenic Escherichia coli LY01. Biotechnol Bioeng 66:203–210. doi:10.1002/(sici)1097-0290(1999)66:4<203::aid-bit1>3.0.co;2-# 10578090

[35] Suzuki K, Ueda M, Yuasa M, Nakagawa T, Kawamukai M, Matsuda H. 1994. Evidence that Escherichia coli ubiA product is a functional homolog of yeast COQ2, and the regulation of ubiA gene expression. Biosci Biotechnol Biochem 58:1814–1819. doi:10.1271/bbb.58.1814 7765507

[36] Kwon O, Druce-Hoffman M, Meganathan R. 2005. Regulation of the ubiquinone (coenzyme Q) biosynthetic genes ubiCA in Escherichia coli. Curr Microbiol 50:180–189. doi:10.1007/s00284-004-4417-1 15902464

[37] Lewis LA, Li KB, Gousse A, Pereira F, Pacheco N, Pierre S, Kodaman P, Lawson S. 1991. Genetic and molecular analysis of spontaneous respiratory deficient (res^-^) mutants of Escherichia coli K-12. Microbiol Immunol 35:289–301. doi:10.1111/j.1348-0421.1991.tb01558.x 1943842

[38] Wang J, Feng JA. 2003. Exploring the sequence patterns in the α‐helices of proteins. Protein Eng Des Sel 16:799–807. doi:10.1093/protein/gzg101 14631069

[39] Morales-Laverde L, Trobos M, Echeverz M, Solano C, Lasa I. 2022. Functional analysis of intergenic regulatory regions of genes encoding surface adhesins in Staphylococcus aureus isolates from periprosthetic joint infections. Biofilm 4:100093. doi:10.1016/j.bioflm.2022.100093 36408060 PMC9667196

[40] Khademi SMH, Sazinas P, Jelsbak L. 2019. Within-host adaptation mediated by intergenic evolution in Pseudomonas aeruginosa. Genome Biol Evol 11:1385–1397. doi:10.1093/gbe/evz083 30980662 PMC6505451

[41] Euro L, Belevich G, Verkhovsky MI, Wikström M, Verkhovskaya M. 2008. Conserved lysine residues of the membrane subunit NuoM are involved in energy conversion by the proton-pumping NADH:ubiquinone oxidoreductase (Complex I). Biochim Biophys Acta 1777:1166–1172. doi:10.1016/j.bbabio.2008.06.001 18590697

[42] Wikström M. 1984. Two protons are pumped from the mitochondrial matrix per electron transferred between NADH and ubiquinone. FEBS Lett 169:300–304. doi:10.1016/0014-5793(84)80338-5 6325245

[43] Bräuer L, Brandt W, Schulze D, Zakharova S, Wessjohann L. 2008. A structural model of the membrane-bound aromatic prenyltransferase UbiA from E. coli. Chembiochem 9:982–992. doi:10.1002/cbic.200700575 18338424

[44] Cooper CJ, Krishnamoorthy G, Wolloscheck D, Walker JK, Rybenkov VV, Parks JM, Zgurskaya HI. 2018. Molecular properties that define the activities of antibiotics in Escherichia coli and Pseudomonas aeruginosa. ACS Infect Dis 4:1223–1234. doi:10.1021/acsinfecdis.8b00036 29756762 PMC6449051

[45] Liang P, Fang X, Hu Y, Yuan M, Raba DA, Ding J, Bunn DC, Sanjana K, Yang J, Rosas-Lemus M, Häse CC, Tuz K, Juárez O-O. 2020. The aerobic respiratory chain of Pseudomonas aeruginosa cultured in artificial urine media: role of NQR and terminal oxidases. PLoS One 15:e0231965. doi:10.1371/journal.pone.0231965 32324772 PMC7179901

[46] Yang E, Yao Y, Liu Y, Sun Z, Shi T, Pan Y, Gao S-S, Xu X, Ma G, Liu G. 2023. A gatekeeper residue controls aromatic acceptor specificity of the PHB-type UbiA prenyltransferases. ACS Catal 13:13717–13728. doi:10.1021/acscatal.3c04085

[47] Deatherage DE, Barrick JE. 2014. Identification of mutations in laboratory-evolved microbes from next-generation sequencing data using breseq. Methods Mol Biol 1151:165–188. doi:10.1007/978-1-4939-0554-6_12 24838886 PMC4239701

[48] Wessjohann L, Sontag B. 1996. Prenylation of benzoic acid derivatives catalyzed by a transferase from Escherichia coli overproduction: method development and substrate specificity. Angew Chem Int Ed Engl 35:1697–1699. doi:10.1002/anie.199616971

[49] Meganathan R. 2001. Ubiquinone biosynthesis in microorganisms. FEMS Microbiol Lett 203:131–139. doi:10.1111/j.1574-6968.2001.tb10831.x 11583838

[50] Imai Y, Hauk G, Quigley J, Liang L, Son S, Ghiglieri M, Gates MF, Morrissette M, Shahsavari N, Niles S, Baldisseri D, Honrao C, Ma X, Guo JJ, Berger JM, Lewis K. 2022. Evybactin is a DNA gyrase inhibitor that selectively kills Mycobacterium tuberculosis. Nat Chem Biol 18:1236–1244. doi:10.1038/s41589-022-01102-7 35996001 PMC9844538

[51] Gilchrist CLM, Booth TJ, van Wersch B, van Grieken L, Medema MH, Chooi Y-H. 2021. cblaster: a remote search tool for rapid identification and visualization of homologous gene clusters. Bioinform Adv 1:vbab016. doi:10.1093/bioadv/vbab016 36700093 PMC9710679

[52] Letunic I, Bork P. 2021. Interactive tree of life (iTOL) v5: an online tool for phylogenetic tree display and annotation. Nucleic Acids Res 49:W293–W296. doi:10.1093/nar/gkab301 33885785 PMC8265157

[53] Yang Y, Ke N, Liu S, Li W. 2017. Methods for structural and functional analyses of intramembrane prenyltransferases in the UbiA superfamily. Methods Enzymol 584:309–347. doi:10.1016/bs.mie.2016.10.032 28065269 PMC5432130

[54] Madhavi Sastry G, Adzhigirey M, Day T, Annabhimoju R, Sherman W. 2013. Protein and ligand preparation: parameters, protocols, and influence on virtual screening enrichments. J Comput Aided Mol Des 27:221–234. doi:10.1007/s10822-013-9644-8 23579614

[55] Lomize MA, Pogozheva ID, Joo H, Mosberg HI, Lomize AL. 2012. OPM database and PPM web server: resources for positioning of proteins in membranes. Nucleic Acids Res 40:D370–D376. doi:10.1093/nar/gkr703 21890895 PMC3245162

[56] Erhardt H, Steimle S, Muders V, Pohl T, Walter J, Friedrich T. 2012. Disruption of individual nuo-genes leads to the formation of partially assembled NADH:ubiquinone oxidoreductase (complex I) in Escherichia coli. Biochim Biophys Acta 1817:863–871. doi:10.1016/j.bbabio.2011.10.008 22063474

[57] Baba T, Ara T, Hasegawa M, Takai Y, Okumura Y, Baba M, Datsenko KA, Tomita M, Wanner BL, Mori H. 2006. Construction of Escherichia coli K-12 in-frame, single-gene knockout mutants: the Keio collection. Mol Syst Biol 2:2006.0008. doi:10.1038/msb4100050 PMC1681482 16738554

[58] Novo D, Perlmutter NG, Hunt RH, Shapiro HM. 1999. Accurate flow cytometric membrane potential measurement in bacteria using diethyloxacarbocyanine and a ratiometric technique. Cytometry 35:55–63. doi:10.1002/(sici)1097-0320(19990101)35:1<55::aid-cyto8>3.0.co;2-2 10554181

[59] Letunic I, Bork P. 2024. Interactive tree of life (iTOL) v6: recent updates to the phylogenetic tree display and annotation tool. Nucleic Acids Res:gkae268. doi:10.1093/nar/gkae268 PMC11223838 38613393

