# Supplementary material for: Small molecule produced by Photorhabdus interferes with ubiquinone biosynthesis in Gram-negative bacteria
Source: mBio. 2024 Sep 10;15(10):e01167-24. doi: 10.1128/mbio.01167-24 (PMC11481567; doi:10.1128/mbio.01167-24)
Supplement: Supplemental material — Figures S1 to S9; Tables S1 to S6. [file mbio.01167-24-s0001.docx]

**Supplementary**


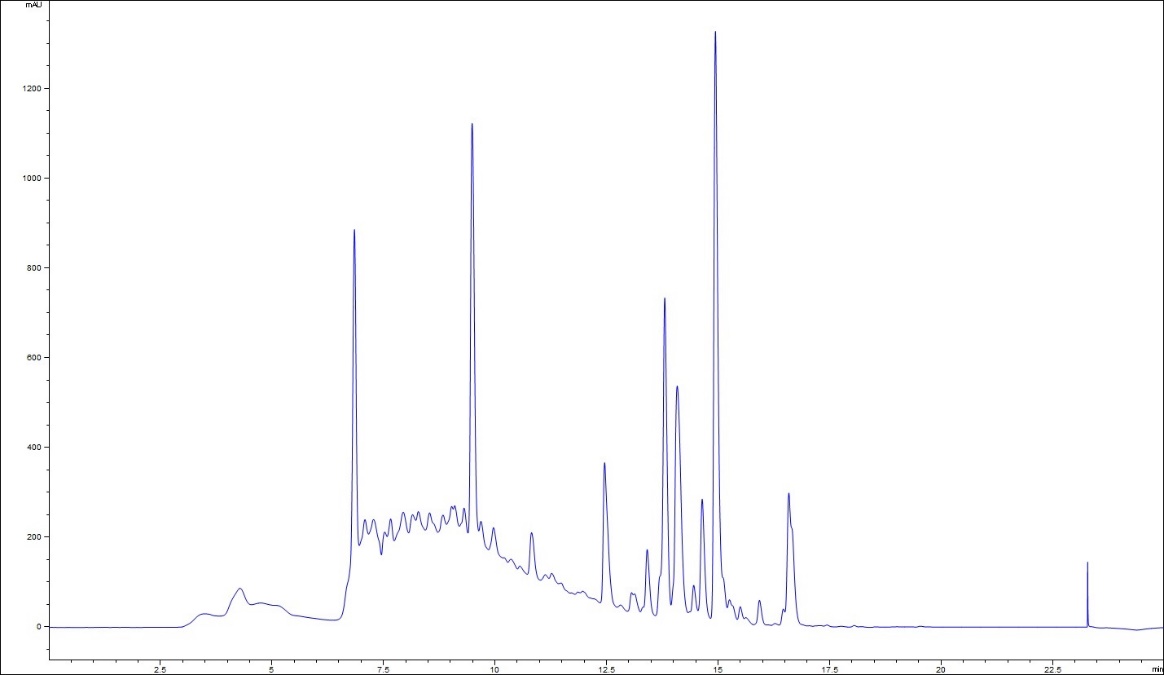


DHB

Fig. S1. RP-HPLC (λ = 254 nm) chromatogram of active fraction post-anion exchange (Q-FF pH 3) from *P. laumondii*. C18 semi-preparative scale HPLC fractionation of eluate from a 200 μL injection concentrated 100x relative to supernatant. Major peaks collected by hand and tested for antibacterial activity against *E. coli* and *S. aureus* bioassay lawn activity assays. Absorbance units (mAU) (y-axis) and time in minutes (x-axis) are shown.


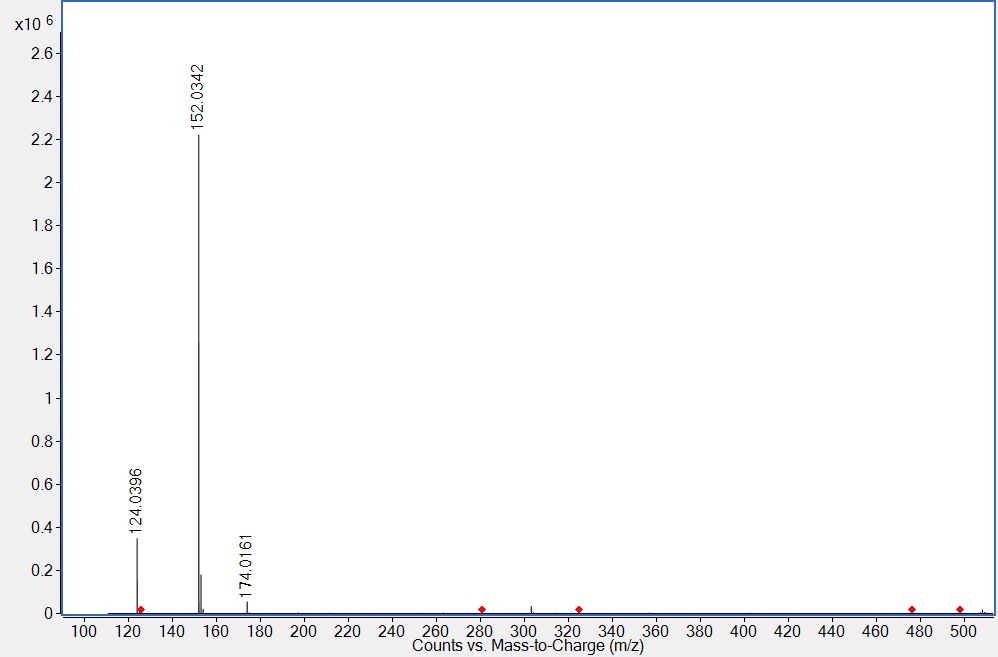


Fig. S2. Mass spectra (MS) of DHB. The exact mass of [M+H]^+^ 152.0342 m/z is determined after injection of the pure compound on the LC-MS. Counts (y-axis) and mass-to-charge (m/z) (x-axis) are shown.


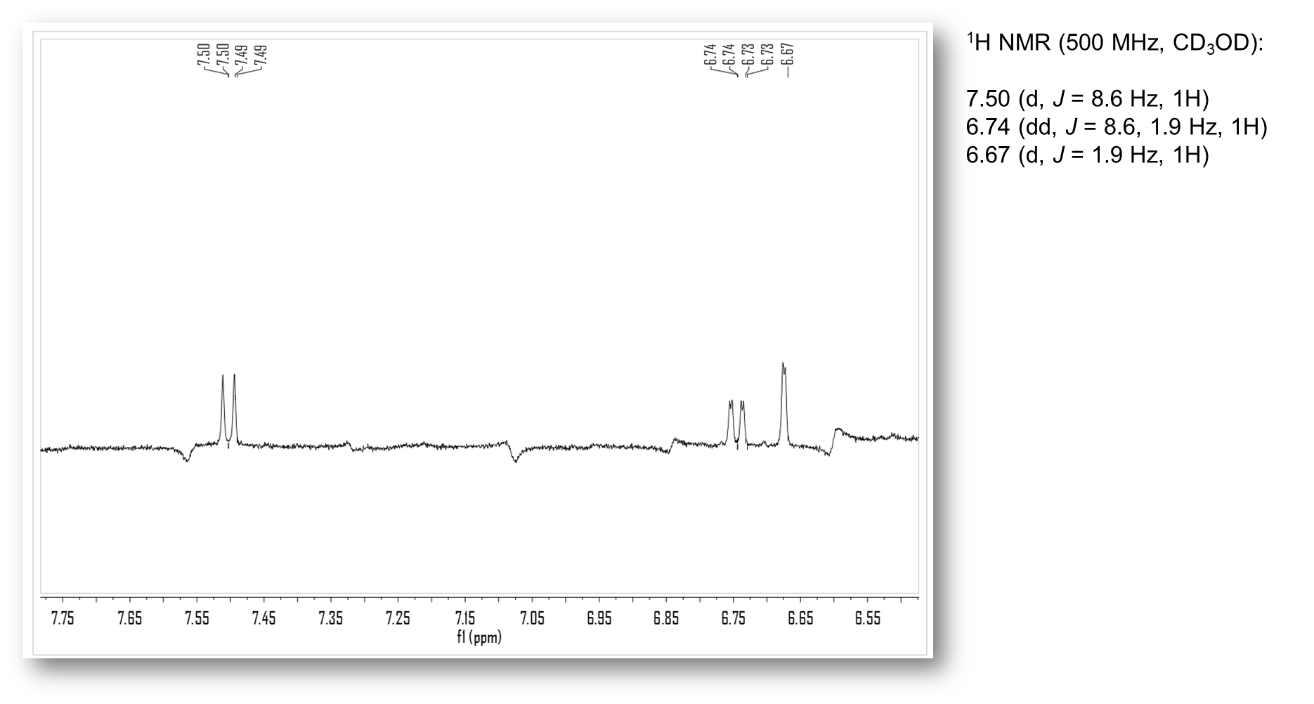


Fig. S3. ^1^H NMR spectrum of the active compound DHB (500 MHz, CD3OD).

Fig. S4. Possible synthetic routes of DHB.

| **NCBI accession (TT01)** | **Name** | ***Photorhabdus laumondii* TT01** | ***Xenorhabdus bovienii* str. kraussei Quebec** | ***Photorhabdus stackebrandtii* DSM 23271** | ***Vibrio* isolate** | ***Vibrio neptunius* KCTC 12702** | ***Chromobacterium piscinae* DSM 23278** |
| --- | --- | --- | --- | --- | --- | --- | --- |
| CAE13294.1 | dhbA | 100 | 88.6 | 68.7 | 75.5 | 75.2 | 63.0 |
| CAE13295.1 | dhbB | 100 | 88.3 | 66.3 | 69.9 | 69.1 | 63.3 |
| CAE13296.1 | dhbC | 99.3 | 79.9 | 40.3 | 40.5 | 40.5 | 36.0 |
| CAE13297.1 | dhbD | 100 | 85.3 | 65.7 | 64.5 | 64.5 | 58.6 |
| CAE13298.1 | dhbE | 100 | 81.1 | n.d. | 57.3 | 58.5 | 52.5 |
| CAE13299.1 | dhbF | 100 | 86.3 | 68.5 | 67.8 | 67.8 | 65.0 |
| CAE13330.1 | dhbG | 100 | 79.7 | 53.1 | 56.6 | 57.3 | 47.0 |

Table S1. Amino acid identity of genes in DHB-like BGCs to their homologs in *P. laumondii* TT01. N.d. indicates no homolog was detected in proximity to the BGC at an identity cutoff of 30%.


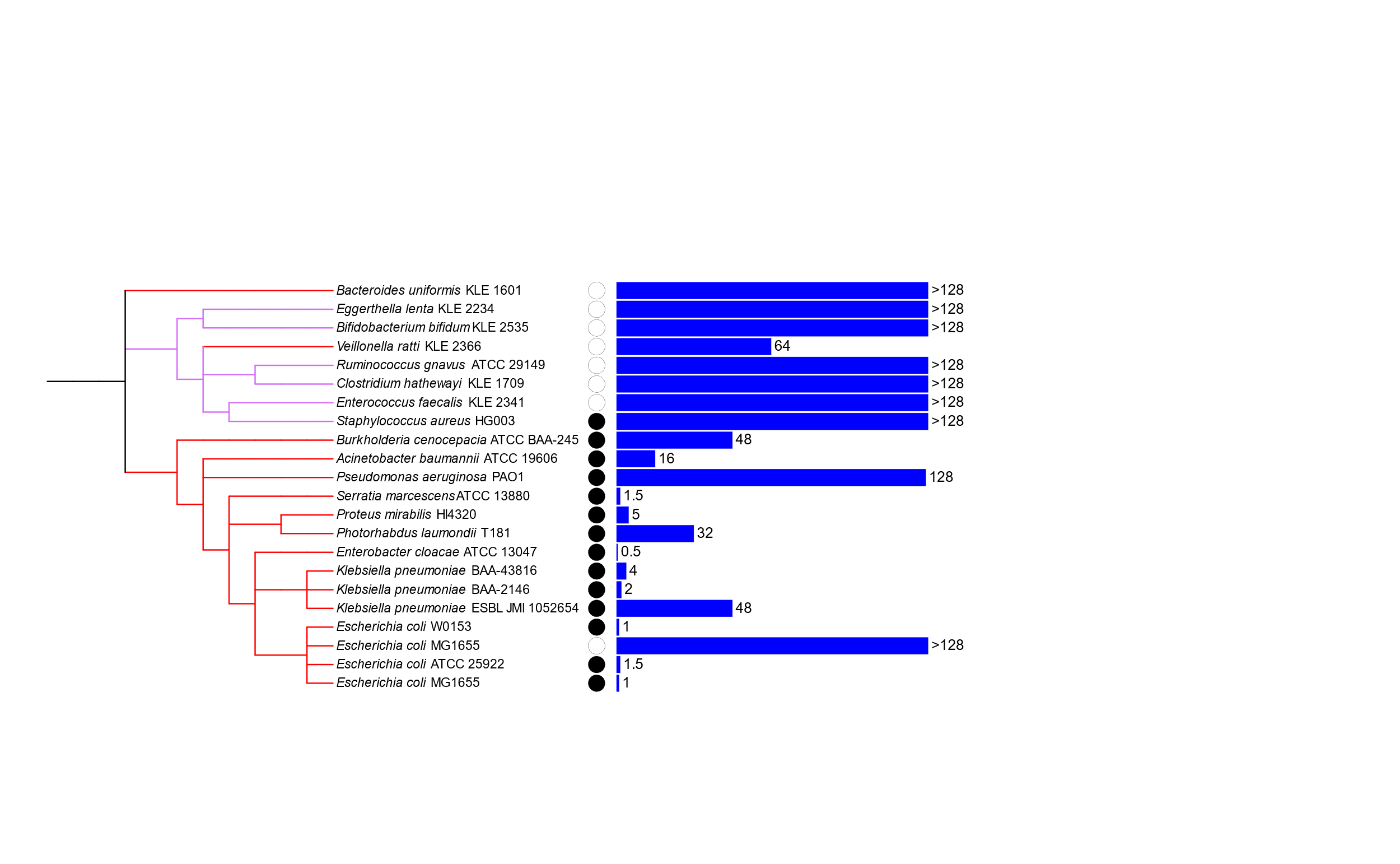


Fig. S5. Phylogenetic organization of anerobic and aerobic MICs. Purple branches denote Gram-positive organisms and red branches represent Gram-negative organisms. White circles indicate MIC testing was performed under anaerobic conditions and black circles denote aerobic testing. All MIC testing was performed in duplicate. Figure created using iTOL (60).


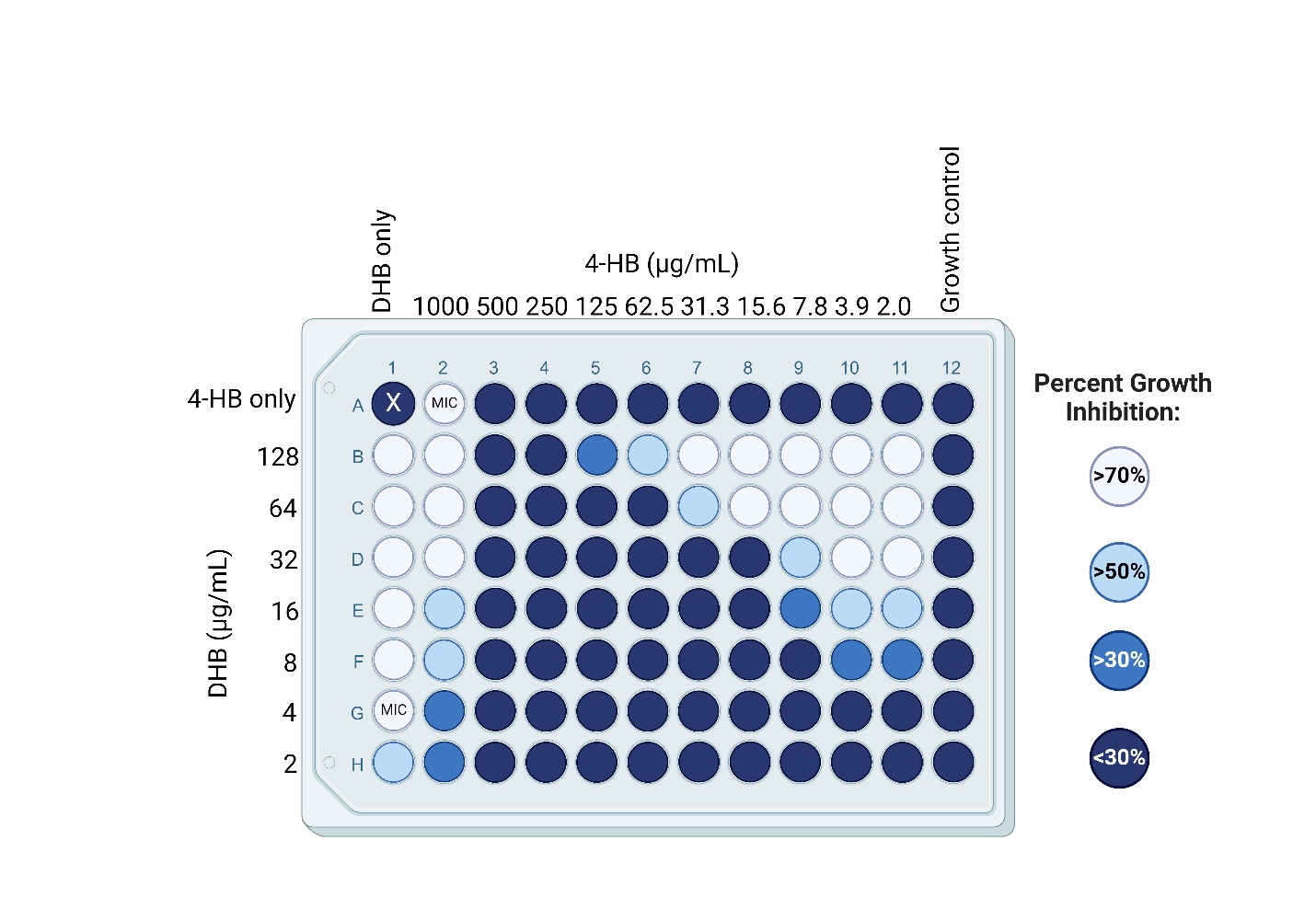


Fig. S6. DHB and 4-hydroxybenzoic acid checkerboard assay in *E. coli* MG1655. Row A contains only 4-HB and column 1 contains only DHB. Column 12 contains *E. coli* only. Increasing inhibition is observed when concentrations of DHB decreases as concentrations of 4-HB increases. An FIC score of 8 indicates an antagonistic relationship.

| *Escherichia coli* MG1655 constructs | MIC (μg ml^-1^) |
| --- | --- |
| Strains | DHB |
| *Escherichia coli* MG1655 (parental) | 1 |
| *ΔaaeB* | 16-64 |
| *pMMB67EH::aaeB* no IPTG | 2 |
| *pMMB67EH::aaeB* 0.1 mM IPTG | 0.5 |
| *pMMB67EH::aaeB* 1 mM IPTG | 0.25 |
| *pMMB67EH* vector only, no IPTG | 1 |
| *pMMB67EH* 0.1 mM IPTG | 1 |
| *pMMB67EH* 1 mM IPTG | 1-2 |

Table S2. MICs of *aaeB* knockout (*ΔaaeB*) and overexpression (*pMMB67EH::aaeB*) strains.

| Strain | DHB MIC (μg ml^-1^) | |
| --- | --- | --- |
| Anaerobic conditions | MHIIB | MOPS + glucose |
| *Escherichia coli* MG1655 (parental) | >128 | >128 |
| *ΔhemB* | >128 | >128 |
| Aerobic conditions |  |  |
| *Escherichia coli* MG1655 (parental) | 1 | >128 |
| *ΔhemB* | >128 | >128 |

Table S3. Mutants lacking *hemB* (porphobilinogen synthase) lose the ability to undergo aerobic respiration. Supplementing minimal media with 0.4% glucose eliminates DHB activity.

Fig. S7. *E. coli* MG1655 *ubiA* and *ubiC* overexpression construct MICs. MIC was measured in duplicate and SD is shown.

Fig. S8. Membrane potential probe DiOC_2_(3) was used for PMF quantification. CCCP dissipates the proton gradient and was used as a positive control. *E. coli* MG1655 cells in early exponential phase in MHIIB medium were treated with 32x MIC DHB at time 0. Fluorescence of individual cells was analyzed by FACS (BD FACS Aria II) for green fluorescence (FITC channel) and red fluorescence (mCherry channel). The ratio of red/green channel shown here is used to determine PMF. Each data point represents the average signal from 10^5^ cells.

| **Organism and genotype** | **DHB MIC (μg/mL)** | **Protein sequence homology (%)** | **Catalytic site 1 homology (%)** | **Catalytic site 2 homology (%)** |
| --- | --- | --- | --- | --- |
| *Escherichia coli* MG1655 | 1 | 100 | 100 | 100 |
| *Escherichia coli* ATCC 25922 | 1-2 | 100 | 100 | 100 |
| *Escherichia coli* W0153 | 1 | 100 | 100 | 100 |
| *Enterobacter cloacae* ATCC 13047 | 0.5 | 91.00 | 100 | 94.12* |
| *Serratia marcescens* NRRL B2544 | 1-2 | 73.10 | 86.96 | 82.35 |
| *Klebsiella pneumoniae* BAA-2146 | 2 | 88.89 | 100 | 100 |
| *Klebsiella pneumoniae* BAA-43816 | 4 | 88.89 | 100 | 100 |
| *Proteus mirabilis* HI4320 | 2-8 | 70.07 | 78.26 | 88.24 |
| *Acinetobacter baumannii* ATCC 19606 | 16 | 49.65 | 78.26 | 70.59 |
| *Pseudomonas aeruginosa* PAO1 | 128 | 53.45 | 82.61 | 70.59 |
| **Pearson correlations to MIC (excluding *P. aeruginosa* PAO1)** | **-** | **-0.85** | **-0.71** | **-0.81** |
| **+ *P. aeruginosa* PAO1** | **-** | **-0.60** | **-0.43** | **-0.64** |
| **+ *ΔubiA::ubiA* PAO1** | **-** | **-0.78** | **-0.70** | **-0.71** |

*minor V to L swap

Table S4. UbiA sequence and catalytic site homology among species.

| Bacterial strains | DHB MIC (μg ml^-1^) |
| --- | --- |
| *P. aeruginosa* PAO1 | 128 |
| *P. aeruginosa* PΔ6 | 64 |
| *P. aeruginosa* PAO1-Pore no IPTG | 128 |
| *P. aeruginosa* PAO1-Pore 0.1 mM IPTG | 128 |
| *P. aeruginosa* PAO1-Pore 1 mM IPTG | 128 |
| *E. coli ΔubiA pMMB67EH::ubiA* MG1655 no IPTG | 0.5-1 |
| *E. coli ΔubiA pMMB67EH::ubiA* MG1655 0.1 mM IPTG | 0.25-0.5 |
| *E. coli ΔubiA pMMB67EH::ubiA* MG1655 1 mM IPTG | 0.25 |
| *E. coli ΔubiA pMMB67EH::ubiA* PAO1 no IPTG | 4 |
| *E. coli ΔubiA pMMB67EH::ubiA* PAO1 0.1 mM IPTG | 8 |
| *E. coli ΔubiA pMMB67EH::ubiA* PAO1 1 mM IPTG | 4 |
| *E. coli ΔubiA pMMB67EH* no IPTG | >128 |
| *E. coli ΔubiA pMMB67EH* 0.1 mM IPTG | >128 |
| *E. coli ΔubiA pMMB67EH* 1 mM IPTG | >128 |

Table S5. MICs of a *ubiA* knockout strain (*ΔubiA*) complemented with either *E. coli* (*ubiA* MG1655) or *P. aeruginosa* (*ubiA* PAO1). All MIC testing was performed in duplicate.

a.


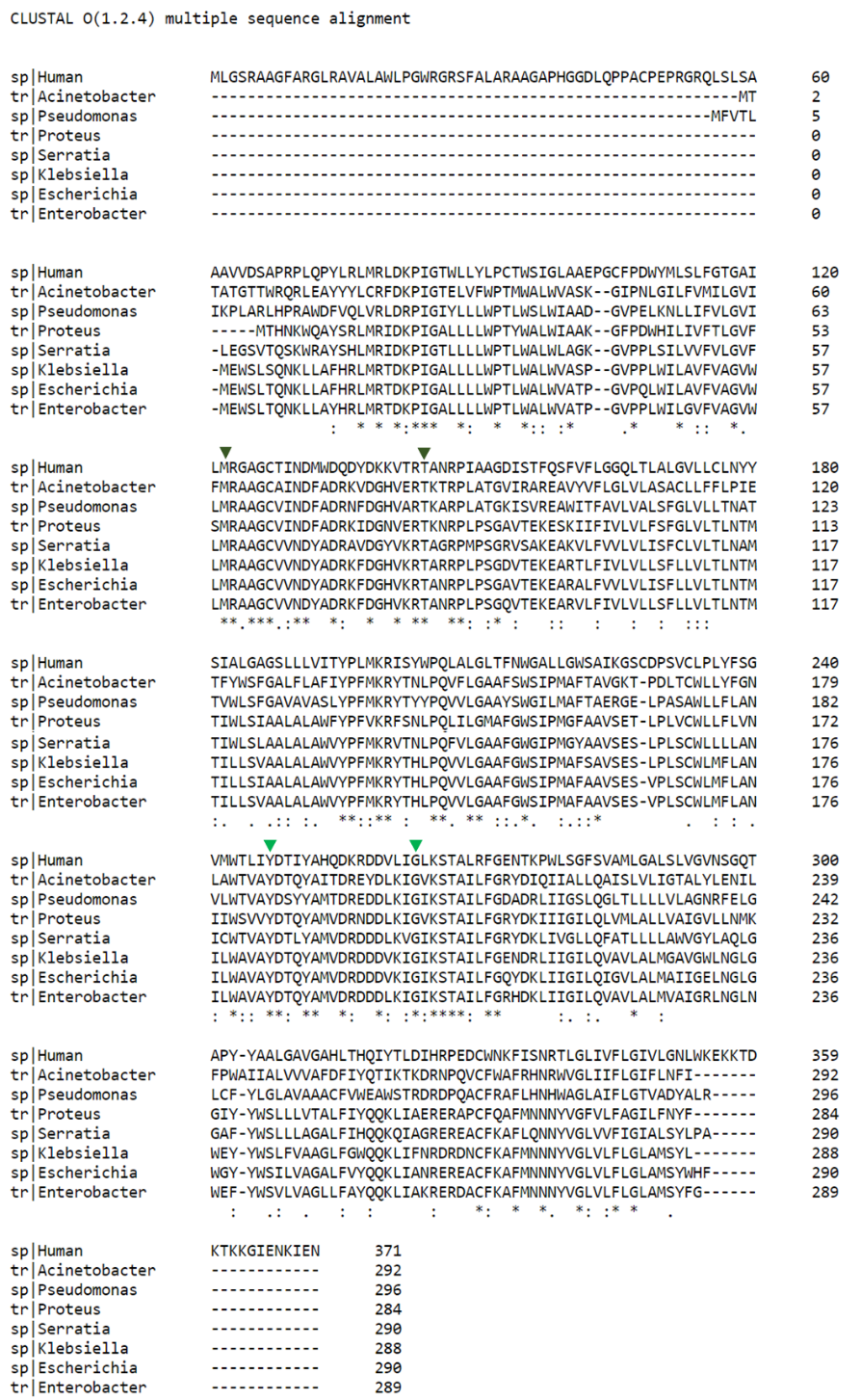


b.


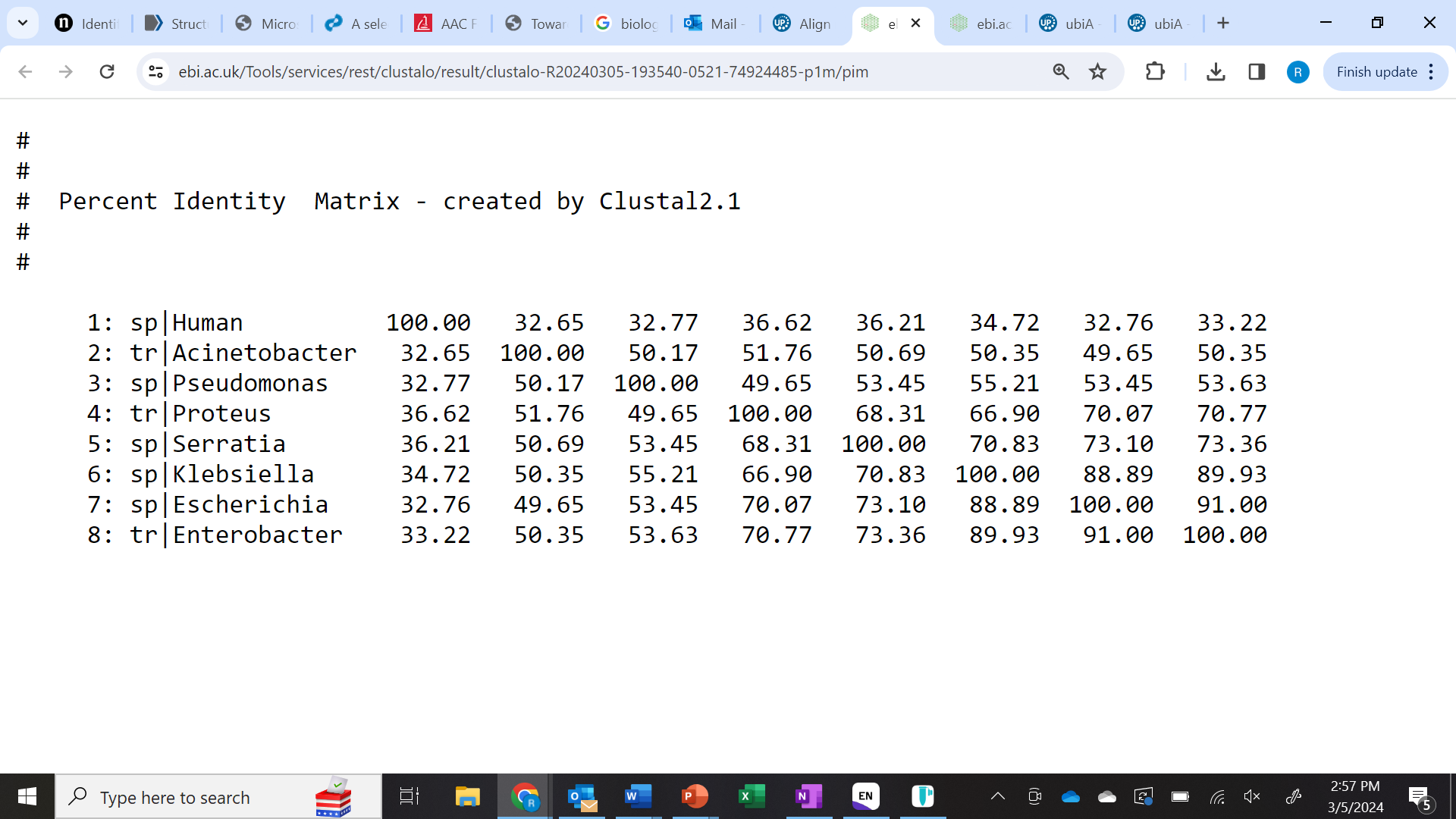


Fig. S9. a. Sequence alignment and b. percent identity of UbiA in *E. coli* (UniProt acc. P0AGK1)*, E. cloacae* (UniProt acc. A0A0H3CDF7)*, K. pneumoniae* (GenBank, ATCC BAA-2146)*, S. marcescens* (GenBank, ATCC 13880)*, P. mirabilis* (UniProt acc. B4EYR6)*, P. aeruginosa* (UniProt acc. Q9HTK0)*, A. baumannii* (UniProt acc. D0CC52)*,* and human homolog COQ2 (UniProt acc. Q96H96). Dark green and light green arrows bound catalytic sites 1 and 2, respectively.

| *Escherichia coli* MG1655 *ΔubiA* aerobic colony sequencing | | |
| --- | --- | --- |
| Colony | Location | Mutation |
| 1 | *nuoN*374 | Non-synonymous substitution  Alanine 🡪 Glutamic acid |
| 2 | *nuoM*114 | Non-synonymous substitution  Glycine 🡪 Valine |
| 3 | *nuoN*427 | Non-synonymous substitution  Arginine 🡪 Serine |
| 4 | *nuoG*301 | Non-synonymous substitution  Glycine 🡪 Aspartic acid |

Table S6. Mutations in *ΔubiA* that allowed for aerobic colony growth.
